# A multi-country survey of public support for food policies to promote healthy diets: Findings from the International Food Policy Study

**DOI:** 10.1186/s12889-019-7483-9

**Published:** 2019-09-02

**Authors:** Janelle Kwon, Adrian J. Cameron, David Hammond, Christine M. White, Lana Vanderlee, Jasmin Bhawra, Gary Sacks

**Affiliations:** 1 Global Obesity Centre (GLOBE), School of Health and Social Development, Deakin University, Geelong, Australia; 2 School of Public Health and Health Systems, University of Waterloo, Waterloo, Canada

**Keywords:** Public opinion, Healthy diet, Public policy, Global health

## Abstract

**Background:**

Poor diet is a significant contributor to the burden of global disease. There are numerous policies available to address poor diets; however, these policies often require public support to encourage policy action. The current study aimed to understand the level of public support for a range of food policies and the factors associated with policy support.

**Methods:**

An online survey measuring support for 13 food policies was completed by 19,857 adults in Australia, Canada, Mexico, the United Kingdom (UK) and the United States (US). The proportion of respondents that supported each policy was compared between countries, and the association between demographic characteristics and policy support was analysed using multivariate logistic regression.

**Results:**

The level of support varied between policies, with the highest support for policies that provided incentives (e.g., price subsidies) or information (e.g., calorie labelling on menus), and the lowest support for those that imposed restrictions (e.g., restrictions on sponsorship of sport events). This pattern of support was similar in all countries, but the level differed, with Mexico generally recording the highest support across policies, and the US the lowest. Several demographic characteristics were associated with policy support; however, these relationships varied between countries.

**Conclusion:**

The results suggest that support for food policies is influenced by several factors related to the policy design, country, and individual demographic characteristics. Policymakers and advocates should consider these factors when developing and promoting policy options.

**Electronic supplementary material:**

The online version of this article (10.1186/s12889-019-7483-9) contains supplementary material, which is available to authorized users.

## Background

It is widely accepted that diet plays an important role in human health [1]. Dietary patterns that do not support good health are characterised by high consumption of saturated fat, salt and free sugars, low consumption of fruits, vegetables and wholegrains, and excess energy intake [1]. Poor diet is a major, and modifiable risk factor for many non-communicable diseases (NCDs) (e.g., cardiovascular diseases, type 2 diabetes and several cancers), and is the leading behavioural risk factor for mortality and the second greatest behavioural risk factor for disease burden globally (as measured by disability adjusted life years) [2].

There is an urgent need to improve dietary patterns and many governments are taking action to promote healthy diets; however, so far these activities have failed to improve diet quality, and in many countries the population is still consuming far too many unhealthy processed foods, and not enough healthy foods such as fruits and vegetables [2]. The lack of dietary improvement is likely because previous interventions have been largely directed at changing individual behaviours (e.g., education or skill building on healthy food preparation and choices), rather than addressing the upstream drivers of poor nutrition that result in the unhealthy food environments we live in [3]. It is widely agreed that the changing food environment, including the increased availability, portion size and marketing, and relatively lower price of energy dense, nutrient poor foods, is the key driver of prevailing poor dietary patterns [4], and that upstream, policy-led solutions are required to counteract these environmental drivers of poor diet [5].

There are a large number of policy solutions that are routinely recommended by health organisations and advocates to improve food environments and promote healthy diets [3, 6]. Despite these recommendations and evidence for the influential role of the food environment, there is lower support among policymakers for policies directed at food environments [7]. Multiple barriers contribute to this limited support and implementation of such policies, including food industry lobbying and relationships with key policymakers, fewer health advocacy resources, and political climates where governments prioritise economic prosperity and favour reduced intervention to encourage market and personal freedom [8, 9]. In addition, there is often a lack of rigorous population-level research evaluating the impact of policies, particularly when they are implemented at a national level, resulting in limited evidence to inform or support government decisions [8]. An unknown or perceived lack of public support may also contribute to the limited range of implemented policies, as governments may be less inclined to implement policies unless they are strongly supported by the voting public [10].

Research examining public support for various food policies has shown that there are high levels of support for education programs and labelling policies, but much lower support for policies that restrict availability or impose financial disincentives [10–14]. Many of these studies also examined the relationship between the demographic characteristics (e.g., age, sex, education) of individuals and policy support; however, the direction and significance of these relationships differed both between policies and between studies. Most prior studies were conducted within individual countries including Canada [14], France [15], the United Kingdom (UK) [16], Australia [17, 18], Netherlands [19], and the United States (US) [20–22], or within the European Union [11, 12, 23]. Thus, it is largely unknown how the social and political environment within each country influences public support, and whether location may contribute to the conflicting findings in previous studies.

The limited cross-country analysis represents a gap in the understanding of policy support, and the opportunities to influence this support. This study aims to address this gap by examining the level of public support for a range of policies, and the demographic factors that may influence this support, both within and between countries.

## Methods

### Study design and participants

Data are from the International Food Policy Study (IFPS) [24], conducted in five countries – Australia, Canada, Mexico, the UK and the US. These countries were chosen because each has begun to implement novel regulations in the areas of food labelling, marketing and taxation, and this repeated cross-sectional study was designed to test the impact of regulation on public policy support.

Participants were recruited through the Nielsen Consumer Insights Global Panel and their partners’ panels, with a random sample of eligible participants invited by email to complete the survey. Individuals aged 18–64 years living within the target countries were eligible to participate. All Canadian participants aged 18–30 years and some respondents aged 31–32 years were recruited separately from the parallel Canada Food Study. Further details on the Canada Food Study are available elsewhere [25].

Data were collected through self-completed web-based surveys in December 2017. The survey questionnaire was developed for the IFPS and collected information on demographic characteristics and various food and food policy-related attitudes, behaviours and knowledge. The questionnaire is available at http://foodpolicystudy.com/methods. Surveys were conducted in English in Australia and the UK, Spanish in Mexico, Spanish or English in the US, and French or English in Canada (based on the participant’s preferred language). Surveys were translated to Spanish and French by third party translation services. Members of the research team who were native in each language independently reviewed the translated surveys. The mean time to complete the survey across all countries was 33 min.

The study was reviewed by and received ethics clearance from a University of Waterloo Research Ethics Committee (ORE# 21460) prior to data collection. Respondents were asked to provide consent before participating and were able to exit the survey at any point. Respondents received remuneration in line with the panel’s standard incentive structure, which included points-based or monetary compensation and/or chances to win prizes. Further details of the study methods are reported elsewhere [24].

### Measures

The current study analysed survey questions related to respondent support for various food policies. Support for 21 food policies was measured, including menu labelling, front-of-pack labelling, food taxation and subsidies, school food policies, and marketing restrictions. The policies were adapted from previous studies [13, 26] and were included based on their potential to support healthy diets, and because they have been considered or implemented by various governments worldwide. Only 13 policies were analysed in the current study, and were chosen based on having closest alignment with recommendations or implemented policy (e.g., a tax on sugary drinks was included but a tax on sugar was not, as a sugary drink tax is more widely recommended [27]). A full list of the 21 policies is available at http://foodpolicystudy.com/methods

Policy support was measured by asking respondents ‘Would you support or oppose a government policy that would require … ’ and completing the question with each of the policies listed in Table 2, with the list shown in a randomised order. Respondents could select either ‘support’, ‘neutral’, ‘oppose’, ‘don’t know’ or ‘refuse to answer’. These responses were re-categorised into a binary variable for analysis, where responses of ‘support’ were categorised as support, and responses of ‘neutral’ and ‘oppose’ were categorised as non-support. Responses of ‘don’t know’ and ‘refuse to answer’ were excluded from the analysis.

Self-reported demographic variables were also measured including age, gender, ethnicity, education, parental status and body mass index (BMI) classification. Education level was categorised as low, medium or high according to country-specific criteria related to the highest level of education completed. Parental status was based on whether the respondent had children (including step or adopted children) aged under 18 years. Height and weight data were used to calculate BMI, which was categorised as underweight (< 18.5 kg/m^2^), normal weight (18.5–24.9 kg/m^2^), overweight (25.0–29.9 kg/m^2^) or obese (≥30.0 kg/m^2^). Age was categorised into five-year age groups, with the exception of the youngest group which included respondents aged 18–24 years. Ethnicity was categorised as ‘majority’ if participants identified themselves as ‘white’, predominantly English-speaking or non-Indigenous (criteria varied according to the most appropriate terminology in each country).

### Data analysis

All statistical analyses were conducted using Stata, Version 15 [28]. Data were weighted with post-stratification sample weights constructed using population estimates from the census in each country based on age group, gender and region. Estimates reported are weighted unless otherwise specified.

The proportion of respondents that supported each of the 13 policies was analysed by country and as a total sample. Logistic regression models were fitted to examine the association between demographic characteristics (country, age, gender, ethnicity and education) and support for each of the policies. This analysis was completed among the total sample, and among each country individually. Covariates included in the model were determined by exploratory univariate logistic regression analysis. Covariates investigated included country, sex, age, education, ethnicity, BMI and parental status. Two-way interactions between country and each of the covariates included in the model were assessed using post-estimation contrast and plots. The final model was adjusted for age, gender, ethnicity and education. Post-estimation contrasts, adjusted for multiple comparisons using a Bonferroni correction, were conducted on all fitted models to determine the overall significance of each covariate included in the model. The logistic regression model fitted among the total sample was repeated with each country as the reference to determine differences in support between countries. The significance level for this analysis was set at *p* < 0.01 to account for multiple comparisons.

## Results

### Sample characteristics

A total of 25,692 respondents completed the survey. After removing ineligible or non-consenting respondents and those that did not pass a data quality check, data from a total of 19,857 respondents in Australia (*n* = 3767), Canada (*n* = 3118), Mexico (*n* = 4057), UK (*n* = 4047) and US (*n* = 4868) were available for analysis. Table 1 describes the sample characteristics, stratified by country. The mean age of respondents across the five countries ranged between 37.1–41.1 years. Each country had approximately equal proportions of male and female respondents. In all countries, the greatest proportion of respondents were classified as of normal weight and of high education.

**Table 1:** Sample demographics, by country (*n* = 19,857)

	Australia, *n* = 3767		Canada, *n* = 3118		Mexico, *n* = 4057		UK, *n* = 4047		US, *n* = 4868	
	Unweighted	Weighted	Unweighted	Weighted	Unweighted	Weighted	Unweighted	Weighted	Unweighted	Weighted
Gender % (n)										
Male	40.0 (1505)	49.6 (1870)	43.9 (1370)	50.2 (1565)	49.7 (2017)	47.8 (1938)	48.7 (1.971)	49.8 (2016)	47.0 (2289)	49.8 (2424)
Female	60.0 (2262)	50.4 (1897)	56.1 (1748)	49.8 (1553)	50.3 (2.040)	52.2 (2119)	51.3 (2.075)	50.2 (2031)	53.0 (2579)	50.2 (2444)
Age Mean (SD)	40.2 (14.6)	40.4 (13.3)	41.9 (15.4)	40.5 (13.7)	33.7 (11.4)	37.1 (12.6)	37.3 (13.3)	40.8 (13.2)	39.1 (14.0)	41.1 (13.5)
Education % (n)										
Low	27.0 (1018)	26.9 (1013)	15.5 (484)	14.1 (440)	18.1 (735)	17.0 (688)	24.6 (996)	25.5 (1033)	19.3 (941)	21.1 (1027)
Medium	35.4 (1334)	35.2 (1324)	34.8 (1084)	34.4 (1072)	12.3 (501)	12.5 (508)	28.4 (1150)	28.3 (1145)	17.7 (863)	19.5 (947)
High	36.9 (1390)	37.1 (1399)	48.9 (1525)	50.7 (1582)	68.4 (2774)	69.4 (2817)	46.1 (1865)	45.3 (1834)	62.5 (3043)	58.9 (2869)
Not stated	0.7 (25)	0.8 (30)	0.8 (25)	0.8 (24)	1.2 (47)	1.1 (45)	0.9 (36)	0.9 (35)	0.4 (21)	0.5 (24)
Ethnicity % (n)										
Majority	83.2 (3134)	81.6 (3076)	70.0 (2184)	67.2 (2096)	86.0 (3489)	86.0 (3489)	87.8 (3.552)	88.4 (3579)	66.9 (3255)	65.0 (3166)
Minority	16.1 (607)	17.5 (661)	27.8 (868)	39.5 (950)	12.5 (509)	12.7 (515)	11.3 (456)	10.4 (420)	32.2 (1568)	34.0 (1656)
Not stated	0.7 (26)	0.8 (31)	2.1 (66)	2.3 (72)	1.5 (61)	1.3 (52)	1.0 (39)	1.2 (48)	0.9 (45)	0.9 (46)
BMI % (n)										
Underweight	3.1 (118)	2.8 (104)	2.8 (87)	2.9 (91)	2.6 (106)	2.5 (100)	3.9 (156)	3.6 (145)	2.4 (115)	2.8 (138)
Normal weight	36.0 (1356)	36.5 (1374)	39.6 (1235)	40.2 (1255)	42.8 (1737)	40.8 (1655)	33.0 (1337)	31.4 (1270)	37.5 (1827)	35.4 (1722)
Overweight	25.0 (941)	25.3 (954)	27.8 (868)	27.4 (854)	31.5 (1277)	33.1 (1341)	20.2 (819)	21.8 (881)	29.5 (1436)	30.4 (1479)
Obese	20.3 (765)	18.8 (707)	18.3 (571)	17.4 (543)	16.0 (649)	17.1 (692)	11.6 (470)	12.5 (505)	22.1 (1078)	22.0 (1073)
Missing	15.6 (587)	16.6 (628)	11.4 (357)	12.0 (376)	7.1 (288)	6.6 (269)	31.2 (1265)	30.8 (1248)	8.4 (412)	9.3 (455)

Sample weights constructed using population estimates from the census in each country based on age group, gender and region

### Public support for food policies

The proportion of respondents that supported each of the policies is reported in Table 2. The level of support varied widely between different policies, ranging between 34.7–68.2% across the total sample. The most supported policy among the total sample was ‘subsidies to reduce the price of fresh fruit and vegetables’ and the least supported policy was ‘a ban on marketing all food and beverages to children’. These policies also received the highest and lowest levels of support among Australian, UK and Mexican respondents when data were analysed separately by country. Among US and Canadian respondents, the most supported policy was ‘calorie amounts on menus of chain restaurants’, and the least supported policy was ‘restriction on sponsorship of sporting events and teams by food companies such as Coca Cola and McDonalds’.

**Table 2:** Weighted proportion of respondents that supported policies in the total sample and by country

	Total *n* = 19,857 %	Australia *n* = 3767 %	Canada *n* = 3118 %	Mexico *n* = 4057 %	UK *n* = 4047 %	US *n* = 4868 %
Subsidies to reduce the price of fresh fruit and vegetables	68.2	68.9^a^	70.4^b^	78.4^a,b,c^	66.5^c^	59.3^a,b,c^
Calorie amounts on menus of chain restaurants^	65.4	60.5^a,b^	70.9^a,c,d^	73.8^b,e,f^	60.6^c,e^	62.6^d,f^
A maximum limit on salt levels in pre-packaged foods	61.4	60.4^a^	64.0^b^	73.6^a,b^	64.0^a,b^	48.5^a,b^
A ban on marketing unhealthy food and beverages to children^	57.2	56.9^a^	61.4^b^	68.1^a,b,c^	59.4^c^	43.5^a,b,c^
Requiring water or milk as the default drink in children’s fast food meal deals	56.0	54.7^a^	55.2^b^	72.2^a,b,c^	52.6^c^	46.4^a,b,c^
Taxes on sugary drinks IF the money was spent on subsidising healthy food^	51.6	48.6^a^	51.8^b^	66.3^a,b^	56.5^a,b^	37.2^a,b^
Taxes on sugary drinks^	42.7	41.8^a^	40.7^b^	53.8^a,b^	49.1^a,b^	30.0^a,b^
Restrictions on maximum size (e.g., max of 375 mL) of single serve soft drink	42.3	43.3^a^	40.9^b^	56.6^a,b,c^	41.0^c^	31.0^a,b,c^
Zoning to restrict the number of fast food restaurants near schools	41.2	44.4^a,b^	35.7^a,b^	49.3^a^	48.4^b^	28.3^a,b^
Taxes on foods with high sugar	41.0	38.5^a,b^	36.1^a^	55.8^a,b^	46.5^a,b^	27.9^b^
A ban on toys, vouchers and competitions as part of children’s meals at fast food restaurants	37.9	42.9^a^	36.2^a,b,c^	43.8^b^	42.8^c^	25.9^a,b,c^
Restriction on sponsorship of sporting events and teams by food companies such as Coca Cola and McDonalds	37.3	39.8^a^	29.7^a^	49.5^a^	43.4^a^	23.3^a^
A ban on marketing all food and beverages to children	34.7	37.8^a^	36.1^b^	40.6^b^	37.8^c^	24.0^a,b,c^

Sample weights constructed using population estimates from the census in each country based on age group, gender and region

Values within rows with the same letters denotes statistically significant different levels of support between those countries at the *p* < 0.01 level (adjusted for age, sex, education and ethnicity)

Policies sorted according to total level of support (in decreasing order)

^denotes policies not measured in Canadian respondents aged 18–30 years. Percentages reported are for adults ages 30 and over

There were several differences in the level of policy support between countries (Table 2). All policies displayed statistically significant differences in policy support between at least two countries (at the *p* < 0.01 level), although these between-country differences differed across the various policies. For example, support for ‘a ban on toys, vouchers and competitions as part of children’s meals at fast food restaurants’ was significantly different between Australia (42.9%) and the US (25.9%), but no such difference was observed for ‘calorie amounts on menus of chain restaurants’, with 60.5% of Australian respondents and 62.6% of US respondents supporting the policy. A significant interaction was observed for most policies between country and sex, and country and age. However, as the main effects were the primary interest in this study, the analysis of differences in support between countries was not adjusted for these interactions.

**Fig. 1:**
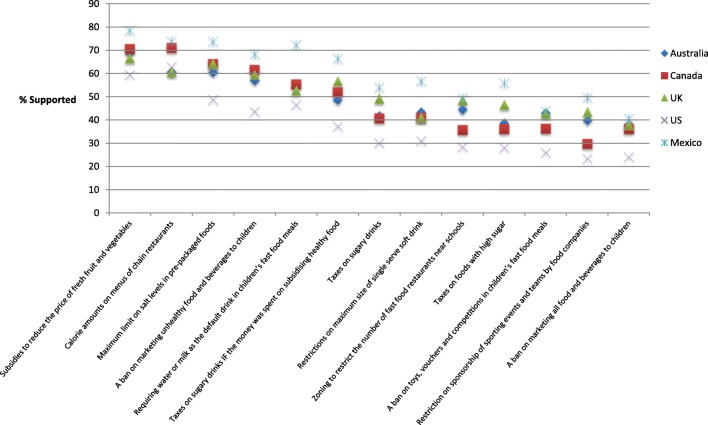
Weighted proportion of respondents that support food policies by country (n = 19,857). Sample weights constructed using population estimates from the census in each country based on age group, gender and region

Figure 1 displays the level of support for the 13 policies in each country. Generally, respondents from Mexico reported the highest level of support and respondents from the US reported the lowest level of support across most policies.

### Demographic characteristics and support for food policies

Results of the logistic regression model fitted for support of the 13 policies across the total sample and in each country are provided in Additional file 1: Table S1, Additional file 2: Table S2, Additional file 3: Table S3, Additional file 4: Table S4, Additional file 5: Table S5, Additional file 6: Table S6. The relationship between demographic characteristics and support for food policies varied between countries. For example, while Australian and US female respondents were more likely than males to support almost all policies, female respondents in Canada were more likely to support only some policies, and there was no significant difference in support between males and females among Mexican respondents for most policies. Older respondents were more likely than respondents aged 18–24 years to support most policies in the Australian, Mexican and UK samples. No association between age and policy support was found among US respondents. Respondents classified as ‘high’ education were more likely than those classified as ‘low’ education to support most policies among Australian and UK respondents, and some policies among US and Canadian respondents. There was generally no association between education and policy support in the Mexican sample. Respondents of minority ethnicity were more likely than respondents of majority ethnicity to support most policies among US respondents. Ethnicity was associated with few policies in each of the remaining countries, although the direction of the relationship varied between countries and between policies.

## Discussion

This study investigated public support for food policies, with a focus on the level and determinants of support within and between countries. The results of this study suggest that policy support is variable and may be influenced by several factors. The level of policy support differed considerably, both between policies and between countries. Several demographic characteristics were also associated with policy support, although this relationship differed between countries.

### Public support for food policies

Public support was highest for policies that provide incentives (e.g., price subsidies), or information (e.g., calorie labelling on menus) and lowest for policies that impose restrictions (e.g., restrictions on sponsorship of sport events). This trend of less support for more restrictive policies, and the order of least to most supported policies was generally consistent in all countries. For example, ‘a ban on marketing all food and beverages to children’ was one of the least supported policies in all countries, and ‘subsidies to reduce the price of fresh fruit and vegetables’ was either the most or the second most supported policy in all countries. This trend also corresponds with previous studies that reported lower support for more ‘intrusive’ policies [10, 14, 19, 23], which according to the Nuffield Council on Bioethics [29], are policies that interfere with individual, population or business liberties.

These findings highlight a key challenge for policymakers, whereby many of the potentially more potent policy options aimed at addressing the underlying drivers of poor diet are also less likely to be supported by the public since they typically involve more intrusive restrictions [30]. Despite this paradox, real world examples demonstrate that policies that are typically considered intrusive are being successfully implemented, such as the introduction of sugary drink taxation policies in several cities and countries [31]. Such examples are encouraging and suggest opportunities exist to implement policies targeted at the drivers of poor diet; however, further research is required to understand the factors that facilitate implementation of more intrusive policy options and the role of public support in such policy action.

It is also important to note that although more intrusive policies received lower support, often this was not accompanied by strong opposition as there was a moderate to large proportion of neutral responses to most policies. For example, while only 37.9% of respondents across all countries supported ‘a ban on the use of toys, vouchers or competitions as part of children’s meals at fast food restaurants’, 42.5% were neutral and only 19.6% were opposed (Additional file 7: Table S7). This finding suggests that policymakers and advocates should consider not only the level of support, but also the level of opposition when evaluating policy feasibility.

### Public support for policies between countries

The results of the current study suggest that country of residence influences policy support levels, with Mexican respondents reporting the highest level of support for all policies across all countries, and US respondents the lowest level of support for almost all policies. Stok et al. [23] similarly found varying levels of support for food policies across four European countries, and suggested that the observed differences may be a result of the degree to which countries exhibited an individualist culture. Individualist countries value self-reliance and prioritise personal goals, while collectivist countries view individuals as a collective community and place the overall wellbeing of the group above personal gains [32]. Due to the emphasis on personal responsibility, individualist countries are more likely to attribute personal behaviours to the individual (e.g., poor dietary choices) rather than the environment (e.g., availability and promotion of unhealthy foods) [33]. Such views may translate into a preference for personal, rather than government, intervention on dietary choices, and may explain the lower support for government introduced food policies. Individualist countries may also be more likely to view government policy as an interference on personal freedom and frame such intervention under the ‘nanny state’ narrative [34]. Nanny state framing, often proffered by the food industry [35], may exaggerate existing individualist beliefs and consequently reduce support. This has been observed in the individualist US, where the 2012 announcement for a restriction on sugary drink portion sizes in New York City was met with ‘nanny state’ declarations by media and lobbyists [36], and strong policy opposition by the public [37].

The findings of the current study generally align with the notion that policy support is influenced by country-level individualist or collectivist beliefs, as we found the lowest support among respondents from the individualist US, and highest support among respondents from the predominantly collectivist, Mexico. On this basis, it may be expected that the other Western countries of the study, which also have individualist cultures, would report lower policy support similar to that of the US; however, the level of support among Australian, Canadian and UK respondents was often significantly higher than US respondents. This aligns with Greenacre’s [34] suggestion that such beliefs exist on a continuum from individualism to collectivism, rather than a dichotomy. Additionally, Lange and Faulkner [33] note that although Canada is an individualist country, it takes a collectivist approach to health (e.g., universal health care), and thus can be differentiated from the highly individualist-dominant US.

An alternative factor that could also contribute to differences in policy support between countries is the political ideology of each country. For example, Diepveen et.al [10]. reported greater support for tobacco and food policies in countries of authoritarian leadership. In the current study, support was highest in Mexico, which has a long history of authoritarian rule [38]. Authoritarian countries are highly loyal to political leadership and thus may be more trusting and accepting of government intervention, and more likely to support government-led policies [38].

Stage of policy implementation has been suggested as another factor that may influence policy support [10] as the tobacco context suggests that policies, such as smoking bans in public areas and plain tobacco packaging, become more acceptable after they have been implemented [39–42]. This could explain the variation in support for policies between countries, if they are at different stages of the policy cycle. There is some evidence of this in the current study, with support for a sugary drinks tax highest in Mexico, where such a tax has been in effect since 2014 [43]. The second highest level of support for this tax was amongst UK respondents, where such a tax was only months away from coming into effect at the time of the current survey [44]. Additionally, support for ‘calorie amounts on menus of chain restaurants’ was the only policy where US respondents did not report the lowest level of support between countries. Menu labelling has been a topical policy measure in the US for some time due to a prolonged period of independent implementation in various cities and states before introduction of a national policy in 2018 [45]. The US is also the only country in the study that has introduced a menu labelling policy at the national level.

It has been suggested that increased policy support following implementation may be related to the fact that many pre-implementation concerns held by the public do not eventuate, or that the public realises that the policy is having a positive impact once implemented [40]. It is also be possible that as policy development and implementation is conducted over an extended period, this may provide an opportunity to educate the public on the benefits of policy action and increase support through media advocacy [46]. However, there is a risk that food industry may alternatively use this period to promote ‘personal responsibility’ and ‘freedom of choice’ narratives in order to shift media framing and reduce public support for government intervention [47]. For example, the fat tax implemented in Denmark received significant negative media coverage directed at the policy shortfalls, and it is thought that such negative framing was a key driver of the eventual repeal of the policy [48].

It is notable that not all results in the current study support the link between policy implementation and support. Although menu labelling has not been implemented in Mexico, support for this policy was highest among Mexican respondents. This suggests that, in this instance, the social or political country context may more strongly influence support, and highlights the complexity of the multiple factors that may contribute to the overall level of policy support. It should also be recognised that interactions between country and both age and sex were observed, meaning that the observed differences may not apply equally across groups based on age and sex.

### Association between demographic characteristics and policy support

In relation to the influence of demographic characteristics on policy support, the current study found that while demographic characteristics may be associated with support for food policies, the patterns differed between countries. Previous studies similarly found demographic variables to be associated with policy support; however, the direction of these relationships varied across studies. For example, in a survey of French adults [15], older respondents were more likely to support a sugary drink tax, while two surveys conducted in the US [21, 49] found younger respondents were more supportive of such a tax. To our knowledge, this is the first study to analyse the relationship between demographic characteristics and policy support across multiple countries and the finding that this relationship varies between countries may partly explain the conflicting findings of earlier studies. Various explanations have previously been proposed for the greater policy support observed among certain demographic sub-groups, such as the greater health consciousness among women [50] and greater trust in government intervention in older age [10]. Results of the current study could suggest that women may be more health conscious in some countries. However, as this is a new area of investigation, further studies are required to replicate and further understand the influence of demographic characteristics on policy support.

### Strengths and limitations

The key strength of this study is the inclusion of multiple countries for the analysis of policy support. To date, this study is the most comprehensive multi-country investigation of food policy support, both in the diversity of countries and range of policies investigated. It is also the first study to compare the effects of demographic characteristics on policy support within different countries.

The findings should nevertheless be interpreted in light of the study’s limitations. Non-probability-based sampling was used to recruit participants, which means that the sample cannot be considered to be nationally representative. The study sample differed from the general population, with a larger proportion of highly educated participants, and a lower proportion of overweight and obese individuals (according to self-reported BMI) compared to national estimates.

Additionally, although the cross-sectional study design is appropriate for determining the ways in which policy support and its determinants vary between countries, repeated surveys are required to assess how support changes over time, particularly in relation to policy implementation. Future waves of data collection in the IFPS will assist in addressing this limitation.

## Conclusions

Similar to previous studies, results of this study indicate that support is greatest for less intrusive policies, and this is consistent across all countries studied. The level of support, however, differed between countries, and may be a result of social and political differences between countries, particularly country-level individualist or collectivist beliefs. There was also some evidence that stage of policy implementation may be associated with support for policies, with greater support as a policy is developed and implemented. A novel finding of this study was that while demographic characteristics may influence support for food policies, this relationship may vary between countries. Overall, this study identified that policy support is influenced by multiple factors related to the policy, the country and the individual. However, further research is required to understand the interaction between these factors, particularly between countries. Stakeholders invested in promoting and developing food policies should consider this complexity when assessing and developing policy options.

## Additional files

Additional file 1: **Table S1.** Results from the logistic regression model for support of food policies across the total sample (*n* = 19,857) (DOCX 42 kb)

Additional file 2: **Table S2.** Results from logistic regression model for support of food policies among Australian respondents (*n* = 3767) (DOCX 45 kb)

Additional file 3: **Table S3.** Results from logistic regression model for support of food policies among Canadian respondents (*n* = 3118) (DOCX 44 kb)

Additional file 4: **Table S4.** Results from logistic regression model for support of food policies among Mexican respondents (*n* = 4057) (DOCX 44 kb)

Additional file 5: **Table S5.** Results from logistic regression model for support of food policies among UK respondents (*n* = 4047) (DOCX 29 kb)

Additional file 6: **Table S6.** Results from logistic regression model for support of food policies among US respondents (*n* = 4868) (DOCX 45 kb)

Additional file 7: **Table S7.** Weighted proportion of ‘support’, ‘neutral’ and ‘oppose’ responses to each policy in the total sample and by country (DOCX 20 kb)

## Data Availability

The datasets analysed during the current study are available from the corresponding author on reasonable request.

## Abbreviations

BMI: Body Mass Index
IFPS: International Food Policy Study
NCD: Non-communicable disease
UK: United Kingdom
US: United States

## References

[1] World Health Organization (WHO) (2003). Food and Agriculture Organization of the United Nations (FAO). Diet, nutrition and the prevention of chronic diseases: Report of a joint WHO/FAO expert consultation.

[2] GBD 2017 Diet Collaborators (2019). Health effects of dietary risks in 195 countries, 1990–2017: a systematic analysis for the Global Burden of Disease Study 2017. Lancet.

[3] Hawkes C, Jewell J, Allen K (2013). A food policy package for healthy diets and the prevention of obesity and diet-related non-communicable diseases: the NOURISHING framework. Obes Rev.

[4] Story M, Kaphingst KM, Robinson-O'Brien R, Glanz K (2008). Creating healthy food and eating environments: policy and environmental approaches. Annu Rev Public Health.

[5] Swinburn BA, Sacks G, Hall KD, McPherson K, Finegood DT, Moodie ML (2011). The global obesity pandemic: shaped by global drivers and local environments. Lancet.

[6] World Health Organization (2013). Global action plan for the prevention and control of noncommunicable diseases 2013–2020.

[7] Millstone E, Lobstein T (2007). The PorGrow project: overall cross-national results, comparisons and implications. Obes Rev.

[8] Clarke B, Swinburn B, Sacks G (2016). The application of theories of the policy process to obesity prevention: a systematic review and meta-synthesis. BMC Public Health.

[9] Cullerton K, Donnet T, Lee A, Gallegos D (2016). Playing the policy game: a review of the barriers to and enablers of nutrition policy change. Public Health Nutr.

[10] Diepeveen S, Ling T, Suhrcke M, Roland M, Marteau TM (2013). Public acceptability of government intervention to change health-related behaviours: a systematic review and narrative synthesis. BMC Public Health.

[11] Reisch LA, Sunstein CR, Gwozdz W (2017). Beyond carrots and sticks: Europeans support health nudges. Food Policy.

[12] Mazzocchi M, Cagnone S, Bech-Larsen T, Niedźwiedzka B, Saba A, Shankar B (2014). What is the public appetite for healthy eating policies? Evidence from a cross-European survey. Health Economics, Policy and Law.

[13] Raine KD, Nykiforuk CIJ, Vu-Nguyen K, Nieuwendyk LM, VanSpronsen E, Reed S (2014). Understanding key influencers’ attitudes and beliefs about healthy public policy change for obesity prevention. Obesity..

[14] Kongats Krystyna, McGetrick Jennifer Ann, Raine Kim D, Voyer Corinne, Nykiforuk Candace IJ (2019). Assessing general public and policy influencer support for healthy public policies to promote healthy eating at the population level in two Canadian provinces. Public Health Nutrition.

[15] Julia C, Méjean C, Vicari F, Péneau S, Hercberg S (2015). Public perception and characteristics related to acceptance of the sugar-sweetened beverage taxation launched in France in 2012. Public Health Nutr.

[16] Beeken RJ, Wardle J (2013). Public beliefs about the causes of obesity and attitudes towards policy initiatives in Great Britain. Public Health Nutr.

[17] Watson W, Weber M, Hughes C, Wellard L, Chapman K (2017). Support for food policy initiatives is associated with knowledge of obesity-related cancer risk factors. Public Health Research & Practice.

[18] Morley B, Martin J, Niven P, Wakefield M (2012). Public opinion on food-related obesity prevention policy initiatives. Health Promotion Journal of Australia.

[19] Bos C, Lans VI, Van Rijnsoever F, Van Trijp H (2015). Consumer acceptance of population-level intervention strategies for healthy food choices: the role of perceived effectiveness and perceived fairness. Nutrients.

[20] Morain S, Mello MM (2013). Survey finds public support for legal interventions directed at health behavior to fight noncommunicable disease. Health Aff.

[21] Curry L, Rogers T, Williams P, Homsi G, Willett J, Schmitt C (2017). Public attitudes and support for a sugar-sweetened beverage tax in America’s heartland. Health Promot Pract.

[22] Donaldson EA, Cohen JE, Rutkow L, Villanti AC, Kanarek NF, Barry CL (2014). Public support for a sugar-sweetened beverage tax and pro-tax messages in a mid-Atlantic US state. Public Health Nutr.

[23] Stok FM, De Ridder DTD, De Vet E, Nureeva L, Luszczynska A, Wardle J, et al. Hungry for an intervention? Adolescent's ratings of acceptability of eating-related intervention strategies. BMC Public Health. 2016;16(1):5. 10.1186/s12889-015-2665-6 PMC4700578 26729328

[24] Hammond D, White C, Mahamad S (2018). International Food Policy Study: Technical Report – Wave 1 (2017).

[25] Hammond D, White C, Reid J. Canada Food Study: Technical Report – Wave 2 (2017). p. 2019. Available from: http://www.canadafoodstudy.ca/studydocs. Accessed 11 April 2019

[26] Bhawra J, Reid JL, White CM, Hammond D, Vanderlee L, Raine K (2018). Are young Canadians supportive of proposed nutrition policies and regulations? An overview of policy support and the impact of socio-demographic factors on public opinion. Canadian Journal of Public Health.

[27] World Health Organization (2016). Report of the commission on ending childhood obesity.

[28] StataCorp (2017). Stata Statistical Software: Release 15.

[29] Nuffield Council on Bioethics (2007). Public health: ethical issues.

[30] Milio N (1989). Nutrition and health: patterns and policy perspectives in food-rich countries. Soc Sci Med.

[31] Cabrera Escobar MA, Veerman JL, Tollman SM, Bertram MY, Hofman KJ (2013). Evidence that a tax on sugar sweetened beverages reduces the obesity rate: a meta-analysis. BMC Public Health.

[32] Singelis TM, Triandis HC, Bhawuk DPS, Gelfand MJ (1995). Horizontal and vertical dimensions of individualism and collectivism: a theoretical and measurement refinement. Cross-Cult Res.

[33] Lange R, Faulkner G (2012). Support for obesity policy: the effect of perceptions of causes for obesity and national identity in Canada. Open J Prev Med.

[34] Greenacre M (2016). Defending public health policies from objections of paternalism. Univ Western Ontario Med J.

[35] Mialon M, Swinburn B, Sacks G (2015). A proposed approach to systematically identify and monitor the corporate political activity of the food industry with respect to public health using publicly available information. Obes Rev.

[36] Wiley LF, Berman ML, Blanke D (2013). Who's your nanny? Choice, paternalism and public health in the age of personal responsibility. The Journal of Law, Medicine & Ethics.

[37] Grynbaum M, Connolly M (2012). 60% in City oppose Bloomberg’s soda ban. Poll Finds New York Times.

[38] Guarneros-Meza V (2009). Mexican urban governance: how old and new institutions coexist and interact. Int J Urban Reg Res.

[39] Lykke M, Helbech B, Glümer C (2014). Temporal changes in the attitude towards smoking bans in public arenas among adults in the Capital Region of Denmark from 2007 to 2010. Scandinavian Journal of Public Health.

[40] Swift E, Borland R, Cummings KM, Fong GT, McNeill A, Hammond D (2015). Australian smokers’ support for plain or standardised packs before and after implementation: findings from the ITC four country survey. Tob Control.

[41] Cooper J, Borland R, Yong H-H, Hyland A (2010). Compliance and support for bans on smoking in licensed venues in Australia: findings from the international tobacco control four-country survey. Aust N Z J Public Health.

[42] Fong GT, Hyland A, Borland R, Hammond D, Hastings G, McNeill A (2006). Reductions in tobacco smoke pollution and increases in support for smoke-free public places following the implementation of comprehensive smoke-free workplace legislation in the Republic of Ireland: findings from the ITC Ireland/UK survey. Tob Control.

[43] Colchero MA, Rivera-Dommarco J, Popkin BM, Ng SW (2017). In Mexico, evidence of sustained consumer response two years after implementing a sugar-sweetened beverage tax. Health Aff.

[44] HM Treasury, Soft drinks levy comes into effect [press release]. 5 April 2018. Available at: https://www.gov.uk/government/news/soft-drinks-industry-levy-comes-into-effect. Accessed 19 Aug 2019.

[45] Zlatevska N, Neumann N, Dubelaar C (2018). Mandatory calorie disclosure: a comprehensive analysis of its effect on consumers and retailers. J Retail.

[46] Huang TTK, Cawley JH, Ashe M, Costa SA, Frerichs LM, Zwicker L (2015). Mobilisation of public support for policy actions to prevent obesity. Lancet.

[47] Jenkin GL, Signal L, Thomson G (2011). Framing obesity: the framing contest between industry and public health at the New Zealand inquiry into obesity. Obes Rev.

[48] Bødker M, Pisinger C, Toft U, Jørgensen T (2015). The rise and fall of the world's first fat tax. Health Policy.

[49] Rivard C, Smith D, McCann SE, Hyland A (2012). Taxing sugar-sweetened beverages: a survey of knowledge, attitudes and behaviours. Public Health Nutr.

[50] Sainsbury E, Hendy C, Magnusson R, Colagiuri S (2018). Public support for government regulatory interventions for overweight and obesity in Australia. BMC Public Health.

