# Supplementary material for: A multi-country survey of public support for food policies to promote healthy diets: Findings from the International Food Policy Study
Source: BMC Public Health. 2019 Sep 2;19:1205. doi: 10.1186/s12889-019-7483-9 (PMC6721115; doi:10.1186/s12889-019-7483-9)
Supplement: Supplementary file 1 — Table S1. Results from the logistic regression model for support of food policies across the total sample (n = 19,857) (DOCX 42 kb) [file 12889_2019_7483_MOESM1_ESM.docx]

Additional file 1: Table S1 Results from the logistic regression model for support of food policies across the total sample (n=19,857)

|  | Subsidies to reduce the price of fresh fruit and vegetables | Calorie amounts on menus of chain restaurants | A maximum limit on salt levels in pre- packaged foods | A ban on marketing unhealthy food and beverages to children | Water or milk as the default drink in children’s meals | Taxes on sugary drinks if the money was spent on subsidising healthy food | Taxes on sugary drinks | Restrictions on maximum size of single serve soft drink | Zoning to restrict the number of fast food restaurants near schools | Taxes on foods with high sugar | A ban on toys, vouchers and competitions in children’s fast food meals | Restriction on sponsorship of sporting events and teams by food companies | A ban on marketing all food and beverages to children |
| --- | --- | --- | --- | --- | --- | --- | --- | --- | --- | --- | --- | --- | --- |
|  | AOR  (95%CI) | AOR  (95%CI) | AOR  (95%CI) | AOR  (95%CI) | AOR  (95%CI) | AOR  (95%CI) | AOR  (95%CI) | AOR  (95%CI) | AOR  (95%CI) | AOR  (95%CI) | AOR  (95%CI)) | AOR  (95%CI) | AOR  (95%CI) |
| Country (Reference = Australia) | | | | | | | | | | | | | |
| Canada | 1.00 | 1.55*** | 0.98 | 1.13* | 0.91 | 1.05 | 0.87* | 0.85* | 0.62*** | 0.49*** | 0.63*** | 0.58*** | 0.84* |
|  | (0.87-1.15) | (1.38-1.76) | (0.86-1.11) | (1.00-1.27) | (0.80-1.03) | (0.94-1.17) | (0.78-0.98) | (0.75-0.97) | (0.54-0.70) | (0.44-0.56) | (0.56-0.72) | (0.51-0.67) | (0.74-0.96) |
| UK | 0.89 | 0.97 | 1.17** | 1.10 | 0.92 | 1.26*** | 1.31*** | 0.92 | 1.18** | 1.32*** | 0.99 | 1.17** | 0.99 |
|  | (0.80-1.00) | (0.87-1.09) | (1.05-1.30) | (0.98-1.22) | (0.82-1.02) | (1.13-1.40) | (1.18-1.46) | (0.83-1.03) | (1.06-1.31) | (1.19-1.47) | (0.89-1.10) | (1.05-1.31) | (0.89-1.11) |
| US | 0.65*** | 1.06 | 0.58*** | 0.54*** | 0.69*** | 0.58*** | 0.54*** | 0.55*** | 0.45*** | 0.57*** | 0.42*** | 0.42*** | 0.49*** |
|  | (0.58-0.73) | (0.95-1.19) | (0.52-0.65) | (0.48-0.60) | (0.62-0.76) | (0.52-0.64) | (0.48-0.60) | (0.49-0.61) | (0.41-0.51) | (0.51-0.64) | (0.38-0.47) | (0.38-0.47) | (0.43-0.55) |
| Mexico | 1.72*** | 1.79*** | 1.97*** | 1.65*** | 2.25*** | 1.99*** | 1.53*** | 1.72*** | 1.22*** | 2.08*** | 1.06 | 1.48*** | 1.12* |
|  | (1.52-1.95) | (1.59-2.01) | (1.75-2.21) | (1.47-1.85) | (2.01-2.53) | (1.78-2.22) | (1.37-1.71) | (1.54-1.92) | (1.09-1.36) | (1.86-2.33) | (0.95-1.19) | (1.32-1.65) | (1.00-1.26) |
| *p value* | ***<0.001*** | ***<0.001*** | ***<0.001*** | ***<0.001*** | ***<0.001*** | ***<0.001*** | ***<0.001*** | ***<0.001*** | ***<0.001*** | ***<0.001*** | ***0.048*** | ***<0.001*** | ***<0.001*** |
| Sex (Reference = Male) | | | | | | | | | | | | | |
| Female | 1.98*** | 1.55*** | 1.59*** | 1.36*** | 1.43*** | 1.28*** | 1.12** | 1.49*** | 1.16*** | 1.10** | 1.08* | 1.13** | 1.15*** |
|  | (1.83-2.14) | (1.44-1.66) | (1.48-1.72) | (1.27-1.45) | (1.34-1.54) | (1.19-1.37) | (1.05-1.21) | (1.38-1.60) | (1.08-1.25) | (1.03-1.18) | (1.00-1.16) | (1.05-1.22) | (1.07-1.24) |
| *p value* | ***<0.001*** | ***<0.001*** | ***<0.001*** | ***<0.001*** | ***<0.001*** | ***<0.001*** | ***<0.001*** | ***<0.001*** | ***<0.001*** | ***0.007*** | ***<0.001*** | ***<0.001*** | ***<0.001*** |
| Age (Reference = 18-24yrs) | | | | | | | | | | | | | |
| 25 – 29yrs | 1.25** | 1.00 | 1.23** | 1.26*** | 1.08 | 1.29*** | 1.18** | 1.13* | 1.14* | 1.12 | 1.15* | 1.12 | 1.21** |
|  | (1.10-1.42) | (0.89-1.12) | (1.09-1.39) | (1.12-1.41) | (0.95-1.21) | (1.15-1.44) | (1.05-1.32) | (1.01-1.28) | (1.01-1.29) | (0.98-1.25) | (1.02-1.31) | (0.99-1.26) | (1.07-1.37) |
| 30 – 34yrs | 1.06 | 0.95 | 1.17* | 1.30*** | 1.08 | 1.02 | 1.16* | 1.15* | 1.19* | 1.32*** | 1.28** | 1.15 | 1.38*** |
|  | (0.92-1.23) | (0.83-1.09) | (1.02-1.35) | (1.14-1.49) | (0.94-1.24) | (0.90-1.16) | (1.02-1.33) | (1.00-1.33) | (1.03-1.37) | (1.15-1.51) | (1.11-1.49) | (1.00-1.33) | (1.19-1.60) |

Model uses weighted data adjusted for country, sex, age, education and ethnicity. Covariate p values are adjusted for multiple comparisons using a Bonferroni correction. AOR = Adjusted Odds Ratio. Statistically significant differences denoted by *p<0.05, **p<0.01, ***p<0.001.

**Supplemental Table 1** con’t

|  |  |  |  |  |  |  |  |  |  |  |  |  |  |
| --- | --- | --- | --- | --- | --- | --- | --- | --- | --- | --- | --- | --- | --- |
|  | Subsidies to reduce the price of fresh fruit and vegetables | Calorie amounts on menus of chain restaurants | A maximum limit on salt levels in pre-packaged foods | A ban marketing unhealthy food and beverages to children | Water or milk as the default drink in children’s meals | Taxes on sugary drinks if the money was spent on subsidising healthy food | Taxes on sugary drinks | Restrictions on maximum size of single serve soft drink | Zoning to restrict the number of fast food restaurants near schools | Taxes on foods with high sugar | A ban on toys, vouchers and competitions in children’s fast food meals | Restriction on sponsorship of sporting events and teams by food companies | A ban on marketing all food and beverages to children |
|  | AOR  (95%CI) | AOR  (95%CI) | AOR  (95%CI) | AOR  (95%CI) | AOR  (95%CI) | AOR  (95%CI) | AOR  (95%CI) | AOR  (95%CI) | AOR  (95%CI) | AOR  (95%CI) | AOR  (95%CI)) | AOR  (95%CI) | AOR  (95%CI) |
| 35 – 39yrs | 1.33** | 1.11 | 1.46*** | 1.37*** | 1.25** | 1.13 | 1.19* | 1.26** | 1.27** | 1.63*** | 1.47*** | 1.37*** | 1.47*** |
|  | (1.13-1.57) | (0.96-1.29) | (1.25-1.70) | (1.18-1.58) | (1.08-1.46) | (0.98-1.30) | (1.03-1.37) | (1.09-1.47) | (1.09-1.48) | (1.40-1.89) | (1.26-1.72) | (1.18-1.60) | (1.26-1.72) |
| 40 – 44yrs | 1.47*** | 1.18* | 1.61*** | 1.51*** | 1.32** | 1.12 | 1.32*** | 1.14 | 1.39*** | 1.59*** | 1.58*** | 1.30** | 1.52*** |
|  | (1.24-1.74) | (1.01-1.38) | (1.37-1.89) | (1.30-1.76) | (1.12-1.54) | (0.97-1.30) | (1.14-1.53) | (0.98-1.34) | (1.19-1.63) | (1.37-1.86) | (1.35-1.86) | (1.11-1.53) | (1.29-1.79) |
| 45 – 49yrs | 1.36*** | 1.15 | 1.72*** | 1.46*** | 1.29** | 1.16* | 1.18* | 1.23** | 1.32*** | 1.61*** | 1.67*** | 1.35*** | 1.50*** |
|  | (1.15-1.60) | (0.99-1.34) | (1.47-2.02) | (1.26-1.68) | (1.11-1.51) | (1.01-1.33) | (1.02-1.37) | (1.05-1.43) | (1.13-1.54) | (1.39-1.87) | (1.42-1.95) | (1.15-1.57) | (1.28-1.75) |
| 50 – 54yrs | 1.49*** | 1.31** | 2.22*** | 1.73*** | 1.43*** | 1.33*** | 1.36*** | 1.11 | 1.36*** | 1.72*** | 1.77*** | 1.28** | 1.49*** |
|  | (1.26-1.76) | (1.12-1.53) | (1.89-2.62) | (1.49-2.01) | (1.23-1.68) | (1.15-1.53) | (1.17-1.58) | (0.95-1.30) | (1.17-1.59) | (1.48-2.01) | (1.50-2.07) | (1.09-1.50) | (1.26-1.75) |
| 55 – 59yrs | 1.52*** | 1.59*** | 2.12*** | 1.89*** | 1.66*** | 1.32*** | 1.43*** | 1.07 | 1.31*** | 1.76*** | 1.89*** | 1.15 | 1.35*** |
|  | (1.31-1.77) | (1.38-1.83) | (1.83-2.46) | (1.65-2.16) | (1.44-1.92) | (1.15-1.50) | (1.25-1.64) | (0.93-1.24) | (1.13-1.52) | (1.53-2.03) | (1.63-2.20) | (0.99-1.34) | (1.16-1.57) |
| 60 - 64yrs | 1.44*** | 1.68*** | 2.39*** | 2.09*** | 1.73*** | 1.54*** | 1.57*** | 1.39*** | 1.54*** | 2.02*** | 2.17*** | 1.21* | 1.57*** |
|  | (1.23-1.68) | (1.46-1.94) | (2.07-2.77) | (1.83-2.39) | (1.49-1.99) | (1.35-1.75) | (1.37-1.79) | (1.20-1.60) | (1.33-1.77) | (1.76-2.32) | (1.87-2.51) | (1.05-1.41) | (1.35-1.83) |
| *p value* | ***<0.001*** | ***<0.001*** | ***<0.001*** | ***<0.001*** | ***<0.001*** | ***<0.001*** | ***<0.001*** | ***<0.001*** | ***<0.001*** | ***<0.001*** | ***<0.001*** | ***<0.001*** | ***<0.001*** |
| Education (Reference = Low) | | | | | | | | | | | | | |
| Medium | 1.10 | 1.19** | 1.19** | 1.25*** | 1.12* | 1.28*** | 1.35*** | 1.13* | 1.14* | 1.27*** | 1.23*** | 1.13* | 1.16** |
|  | (0.98-1.23) | (1.07-1.32) | (1.07-1.32) | (1.13-1.38) | (1.01-1.25) | (1.16-1.41) | (1.22-1.50) | (1.01-1.25) | (1.03-1.26) | (1.14-1.41) | (1.10-1.37) | (1.01-1.25) | (1.04-1.30) |
| High | 1.01 | 1.42*** | 1.18** | 1.36*** | 1.14** | 1.59*** | 1.61*** | 1.19*** | 1.19*** | 1.42*** | 1.31*** | 1.19*** | 1.24*** |
|  | (0.92-1.12) | (1.30-1.56) | (1.07-1.29) | (1.24-1.49) | (1.03-1.25) | (1.45-1.74) | (1.46-1.76) | (1.09-1.31) | (1.08-1.31) | (1.30-1.56) | (1.19-1.44) | (1.08-1.31) | (1.12-1.37) |
| *p value* | **0.161** | ***<0.001*** | **0.001** | ***<0.001*** | **0.021** | ***<0.001*** | ***<0.001*** | **0.001** | **0.001** | ***<0.001*** | ***<0.001*** | **0.002** | ***<0.001*** |
| Ethnicity (Reference = Majority) | | | | | | | | | | | | | |
| Minority | 0.97 | *0.84**** | 1.10 | 1.11* | 1.11* | 1.07 | 1.14** | 1.33*** | 1.35*** | 1.22*** | 1.27*** | 1.37*** | 1.19** |
|  | (0.87-1.07) | *(0.77-0.92)* | (1.00-1.21) | (1.01-1.21) | (1.01-1.23) | (0.98-1.17) | (1.04-1.25) | (1.21-1.46) | (1.22-1.48) | (1.12-1.34) | (1.15-1.40) | (1.24-1.51) | (1.08-1.31) |
| *p value* | **0.520** | ***<0.001*** | **0.056** | **0.034** | **0.028** | **0.137** | ***<0.001*** | ***<0.001*** | ***<0.001*** | ***<0.001*** | ***<0.001*** | ***<0.001*** | ***<0.001*** |

Model uses weighted data adjusted for country, sex, age, education and ethnicity. Covariate p values are adjusted for multiple comparisons using a Bonferroni correction. AOR = Adjusted Odds Ratio. Statistically significant differences denoted by *p<0.05, **p<0.01, ***p<0.001
