# Supplementary material for: A multi-country survey of public support for food policies to promote healthy diets: Findings from the International Food Policy Study
Source: BMC Public Health. 2019 Sep 2;19:1205. doi: 10.1186/s12889-019-7483-9 (PMC6721115; doi:10.1186/s12889-019-7483-9)
Supplement: Supplementary file 2 — Table S2. Results from logistic regression model for support of food policies among Australian respondents (n = 3767) (DOCX 45 kb) [file 12889_2019_7483_MOESM2_ESM.docx]

Additional file 2: Table S2 Results from logistic regression model for support of food policies among Australian respondents (n=3,767)

|  | Subsidies to reduce the price of fresh fruit and vegetables | Calorie amounts on menus of chain restaurants | A maximum limit on salt levels in pre-packaged foods | A ban on marketing unhealthy food and beverages to children | Water or milk as the default drink in children’s meals | Taxes on sugary drinks if the money was spent on subsidising healthy food | Taxes on sugary drinks | Restrictions on maximum size of single serve soft drink | Zoning to restrict the number of fast food restaurants near schools | Taxes on foods with high sugar | A ban on toys, vouchers and competitions in children’s fast food meals | Restriction on sponsorship of sporting events and teams by food companies | A ban on marketing all food and beverages to children |
| --- | --- | --- | --- | --- | --- | --- | --- | --- | --- | --- | --- | --- | --- |
|  | AOR  (95%CI) | AOR  (95%CI) | AOR  (95%CI) | AOR  (95%CI) | AOR  (95%CI) | AOR  (95%CI) | AOR  (95%CI) | AOR  (95%CI) | AOR  (95%CI) | AOR  (95%CI) | AOR  (95%CI)) | AOR  (95%CI) | AOR  (95%CI) |
| Sex (Reference = Male) | | | | | | | | | | | | | |
| Female | 2.79*** | 1.92*** | 1.99*** | 1.59*** | 1.67*** | 1.50*** | 1.20* | 1.73*** | 1.34*** | 1.21* | 1.16 | 1.26** | 1.25** |
|  | (2.35-3.31) | (1.64-2.25) | (1.70-2.34) | (1.36-1.86) | (1.43-1.94) | (1.29-1.74) | (1.03-1.40) | (1.48-2.03) | (1.15-1.56) | (1.04-1.42) | (0.99-1.36) | (1.08-1.47) | (1.07-1.46) |
| P value | ***<0.001*** | ***<0.001*** | ***<0.001*** | ***<0.001*** | ***<0.001*** | ***<0.001*** | ***0.019*** | ***<0.001*** | ***<0.001*** | ***0.014*** | ***0.060*** | ***0.003*** | ***0.006*** |
| Age (Reference = 18-24yrs) | | | | | | | | | | | | | |
| 25 – 29yrs | 1.19 | 1.08 | 1.17 | 1.17 | 1.08 | 1.29* | 1.07 | 1.03 | 1.19 | 1.09 | 1.09 | 1.07 | 1.07 |
|  | (0.91-1.56) | (0.84-1.39) | (0.91-1.49) | (0.91-1.50) | (0.85-1.39) | (1.00-1.66) | (0.82-1.38) | (0.80-1.33) | (0.92-1.53) | (0.84-1.42) | (0.84-1.42) | (0.83-1.38) | (0.83-1.39) |
| 30 – 34yrs | 0.93 | 0.92 | 1.27 | 1.27 | 1.41* | 0.93 | 0.93 | 1.13 | 1.12 | 1.09 | 0.96 | 0.82 | 1.10 |
|  | (0.67-1.29) | (0.67-1.260 | (0.93-1.74) | (0.93-1.74) | (1.03-1.93) | (0.68-1.27) | (0.67-1.28) | (0.82-1.55) | (0.81-1.54) | (0.79-1.50) | (0.70-1.34) | (0.59-1.14) | (0.80-1.52) |
| 35 – 39yrs | 1.78** | 1.36 | 1.99*** | 1.70** | 1.65** | 1.56* | 1.49* | 1.59** | 1.38 | 1.53* | 1.55* | 1.37 | 1.56* |
|  | (1.21-2.62) | (0.95-1.93) | (1.40-2.82) | (1.20-2.41) | (1.17-2.33) | (1.11-2.19) | (1.05-2.10) | (1.12-2.24) | (0.98-1.95) | (1.08-2.17) | (1.09-2.21) | (0.97-1.94) | (1.09-2.21) |
| 40 – 44yrs | 2.24*** | 1.84** | 2.33*** | 1.90*** | 1.56* | 1.61** | 1.55* | 1.37 | 1.92*** | 1.58** | 2.04*** | 1.34 | 1.86*** |
|  | (1.51-3.31) | (1.30-2.62) | (1.65-3.30) | (1.35-2.66) | (1.11-2.18) | (1.15-2.25) | (1.10-2.17) | (0.98-1.92) | (1.38-2.78) | (1.13-2.22) | (1.45-2.86) | (0.95-1.88) | (1.32-2.61) |
| 45 – 49yrs | 1.94*** | 1.41* | 2.21*** | 2.03*** | 1.65** | 1.62** | 1.51* | 1.71** | 1.78*** | 1.75** | 1.73** | 1.41* | 1.62** |
|  | (1.37-2.75) | (1.03-1.95) | (1.61-3.03) | (1.48-2.78) | (1.21-2.26) | (1.18-2.21) | (1.10-2.08) | (1.25-2.35) | (1.30-2.44) | (1.27-2.41) | (1.26-2.38) | (1.03-1.94) | (1.17-2.23) |
| 50 – 54yrs | 1.57* | 1.31 | 2.72*** | 2.09*** | 1.50* | 1.85*** | 1.68** | 1.27 | 1.69** | 1.95*** | 2.15*** | 1.47* | 1.32 |
|  | (1.11-2.22) | (0.95-1.79) | (1.96-3.78) | (1.53-2.87) | (1.10-2.05) | (1.36-2.51) | (1.23-2.29) | (0.93-1.74) | (1.24-2.30) | (1.43-2.66) | (1.57-2.95) | (1.08-2.01) | (0.96-1.82) |
| 55 – 59yrs | 2.65*** | 2.00*** | 3.71*** | 2.78*** | 2.00*** | 2.06*** | 1.73*** | 1.30 | 2.05*** | 1.83*** | 2.78*** | 1.45** | 1.41* |
|  | (1.94-3.63) | (1.50-2.67) | (2.78-4.95) | (2.10-3.69) | (1.52-2.63) | (1.56-2.71) | (1.31-2.29) | (0.99-1.71) | (1.56-2.70) | (1.38-2.42) | (2.10-3.68) | (1.10-1.92) | (1.07-1.88) |
| 60 - 64yrs | 2.56*** | 2.14*** | 3.59*** | 2.74*** | 2.29*** | 1.95*** | 1.69*** | 1.93*** | 2.10*** | 1.98*** | 2.93*** | 1.35* | 1.51** |
|  | (1.90-3.44) | (1.63-2.81) | (2.72-4.74) | (2.09-3.58) | (1.75-2.99) | (1.49-2.54) | (1.29-2.22) | (1.49-.52) | (1.61-2.74) | (1.51-2.60) | (2.23-3.84) | (1.03-1.76) | (1.15-1.98) |
| P value | ***<0.001*** | ***<0.001*** | ***<0.001*** | ***<0.001*** | ***<0.001*** | ***<0.001*** | ***<0.001*** | ***<0.001*** | ***<0.001*** | ***<0.001*** | ***<0.001*** | ***0.003*** | ***0.001*** |

Model uses weighted data adjusted for country, sex, age, education and ethnicity. Covariate p values are adjusted for multiple comparisons using a Bonferroni correction. AOR = Adjusted Odds Ratio. Statistically significant differences denoted by *p<0.05, **p<0.01, ***p<0.001.

**Supplemental Table 2** con’t

|  | | | | | | | | | | | | | |
| --- | --- | --- | --- | --- | --- | --- | --- | --- | --- | --- | --- | --- | --- |
|  | Subsidies to reduce the price of fresh fruit and vegetables | Calorie amounts on menus of chain restaurants | A maximum limit on salt levels in pre-packaged foods | A ban on marketing unhealthy food and beverages to children | Water or milk as the default drink in children’s meals | Taxes on sugary drinks if the money was spent on subsidising healthy food | Taxes on sugary drinks | Restrictions on maximum size of single serve soft drink | Zoning to restrict the number of fast food restaurants near schools | Taxes on foods with high sugar | A ban on toys, vouchers and competitions in children’s fast food meals | Restriction on sponsorship of sporting events and teams by food companies | A ban on marketing all food and beverages to children |
|  | AOR  (95%CI) | AOR  (95%CI) | AOR  (95%CI) | AOR  (95%CI) | AOR  (95%CI) | AOR  (95%CI) | AOR  (95%CI) | AOR  (95%CI) | AOR  (95%CI) | AOR  (95%CI) | AOR  (95%CI)) | AOR  (95%CI) | AOR  (95%CI) |
| Education (Reference = Low) | | |  |  |  |  |  |  |  |  |  |  |  |
| Medium | 1.23 | 1.21 | 1.33** | 1.22* | 1.21 | 1.46*** | 1.39** | 1.24* | 1.21 | 1.38** | 1.28* | 1.13 | 0.95 |
|  | (0.98-1.52) | (0.99-1.47) | (1.09-1.62) | (1.00-1.49) | (1.00-1.47) | (1.20-1.77) | (1.14-1.69) | (1.01-1.50) | (1.00-1.47) | (1.12-1.69) | (1.05-1.56) | (0.93-1.38) | (0.78-1.16) |
| High | 1.20 | 1.49*** | 1.48*** | 1.47*** | 1.17 | 1.89*** | 1.89*** | 1.35** | 1.43** | 1.93*** | 1.48*** | 1.23* | 1.17 |
|  | (0.96-1.51) | (1.21-1.83) | (1.20-1.82) | (1.20-1.81) | (0.96-1.43) | (1.54-2.32) | (1.54-2.32) | (1.10-1.65) | (1.17-1.75) | (1.57-2.60) | (1.21-1.82) | (1.00-1.52) | (0.96-1.44) |
| P value | ***0.147*** | ***<0.001*** | ***0.001*** | ***0.001*** | ***0.132*** | ***<0.001*** | ***<0.001*** | ***0.015*** | ***<0.001*** | ***<0.001*** | ***0.001*** | ***0.137*** | ***0.076*** |
| Ethnicity (Reference = Majority) | | |  |  |  |  |  |  |  |  |  |  |  |
| Minority | 0.91 | 0.87 | 1.29* | 0.97 | 1.04 | 0.92 | 1.12 | 1.23 | 0.95 | 1.17 | 1.06 | 1.06 | 1.16 |
|  | (0.72-1.15) | (0.70-1.09) | (1.03-1.62) | (0.78-1.21) | (0.84-1.30) | (0.74-1.13) | (0.90-1.39) | (0.99-1.54) | (0.76-1.18) | (0.94-1.45) | (0.85-1.32) | (0.85-1.31) | (0.93-1.45) |
| P value | ***0.434*** | ***0.2172*** | ***0.029*** | ***0.783*** | ***0.707*** | ***0.423*** | ***0.313*** | ***0.059*** | ***0.617*** | ***0.160*** | ***0.597*** | ***0.628*** | ***0.185*** |

Model uses weighted data adjusted for country, sex, age, education and ethnicity. Covariate p values are adjusted for multiple comparisons using a Bonferroni correction. AOR = Adjusted Odds Ratio. Statistically significant differences denoted by *p<0.05, **p<0.01, ***p<0.001.
