# Supplementary material for: A multi-country survey of public support for food policies to promote healthy diets: Findings from the International Food Policy Study
Source: BMC Public Health. 2019 Sep 2;19:1205. doi: 10.1186/s12889-019-7483-9 (PMC6721115; doi:10.1186/s12889-019-7483-9)
Supplement: Supplementary file 3 — Table S3. Results from logistic regression model for support of food policies among Canadian respondents (n = 3118) (DOCX 44 kb) [file 12889_2019_7483_MOESM3_ESM.docx]

Additional file 3: Table S3 Results from logistic regression model for support of food policies among Canadian respondents (n=3,118)

|  | Subsidies to reduce the price of fresh fruit and vegetables^#^ | Calorie amounts on menus of chain restaurants | A maximum limit on salt levels in pre-packaged foods^#^ | A ban on marketing unhealthy food and beverages to children | Water or milk as the default drink in children’s meals^#^ | Taxes on sugary drinks if the money was spent on subsidising healthy food | Taxes on sugary drinks | Restrictions on maximum size of single serve soft drink^#^ | Zoning to restrict the number of fast food restaurants near schools^#^ | Taxes on foods with high sugar^#^ | A ban on toys, vouchers and competitions in children’s fast food meals^#^ | Restriction on sponsorship of sporting events and teams by food companies^#^ | A ban on marketing all food and beverages to children^#^ |
| --- | --- | --- | --- | --- | --- | --- | --- | --- | --- | --- | --- | --- | --- |
|  | AOR  (95%CI) | AOR  (95%CI) | AOR  (95%CI) | AOR  (95%CI) | AOR  (95%CI) | AOR  (95%CI) | AOR  (95%CI) | AOR  (95%CI) | AOR  (95%CI) | AOR  (95%CI) | AOR  (95%CI)) | AOR  (95%CI) | AOR  (95%CI) |
| Sex (Reference = Male) | | | | | | | | | | | | | |
| Female | 2.39*** | 1.57*** | 1.75*** | 1.38*** | 1.50*** | 1.30** | 1.21* | 1.55*** | 1.08 | 1.03 | 1.02 | 1.11 | 1.08 |
|  | (1.91-2.98) | (1.31-1.89) | (1.42-2.15) | (1.17-1.64) | (1.23-1.83) | (1.10-1.53) | (1.02-1.44) | (1.27-1.88) | (0.88-1.32) | (0.85-1.26) | (0.84-1.25) | (0.90-1.37) | (0.89-1.32) |
| P value | ***<0.001*** | ***<0.001*** | ***<0.001*** | ***<0.001*** | ***<0.001*** | ***0.002*** | ***0.027*** | ***<0.001*** | ***0.446*** | ***0.744*** | ***0.812*** | ***0.347*** | ***0.436*** |
| Age (Reference = 18-24yrs) | | | | | | | | | | | | | |
| 25 – 29yrs | - | 0.72 | - | 1.65** | - | 1.42* | 1.38 | - | - | - | - | - | - |
|  |  | (0.49-1.07) |  | (1.14-2.38) |  | (1.00-2.01) | (0.97-1.96) |  |  |  |  |  |  |
| 30 – 34yrs | - | 0.47*** | - | 1.10 | - | 0.73 | 1.35 | - | - | - | - | - | - |
|  |  | (0.32-0.68) |  | (0.78-1.56) |  | (0.52-1.02) | (0.95-1.92) |  |  |  |  |  |  |
| 35 – 39yrs | 1.44 | 0.53** | 1.25 | 0.72 | 1.18 | 0.55** | 1.05 | 1.04 | 1.46 | 0.49*** | 1.10 | 1.13 | 1.22 |
|  | (0.90-2.30) | (0.36-0.78) | (0.88-2.07) | (0.51-1.01) | (0.77-1.81) | (0.40-0.78) | (0.74-1.50) | (0.67-1.62) | (0.93-2.30) | (0.34-0.72) | (0.70-1.73) | (0.72-1.78) | (0.78-1.91) |
| 40 – 44yrs | 1.71* | 0.50*** | 1.85** | 1.00 | 1.44 | 0.60** | 1.22 | 1.39 | 1.10 | 0.82 | 1.10 | 0.91 | 1.10 |
|  | (1.06-2.77) | (0.34-0.73) | (1.18-2.89) | (0.70-1.42) | (0.93-2.22) | (0.42-0.85) | (0.85-1.74) | (0.90-2.17) | (0.69-1.75) | (0.57-1.17) | (0.70-1.74) | (0.57-1.44) | (0.69-1.73) |
| 45 – 49yrs | 1.49 | 0.53** | 1.73* | 0.98 | 1.24 | 0.53*** | 0.93 | 1.51 | 1.12 | 0.84 | 1.11 | 0.85 | 1.16 |
|  | (0.95-2.34) | (0.37-0.76) | (1.14-2.64) | (0.70-1.36) | (0.82-1.87) | (0.38-0.73) | (0.66-1.31) | (0.99-2.30) | (0.72-1.75) | (0.58-1.22) | (0.71-1.73) | (0.54-1.33) | (0.74-1.80) |
| 50 – 54yrs | 1.42 | 0.59** | 2.27*** | 1.09 | 1.41 | 0.63** | 1.05 | 1.16 | 1.13 | 0.80 | 1.27 | 0.75 | 1.14 |
|  | (0.92-2.20) | (0.41-0.85) | (1.49-3.47) | (0.78-1.51) | (0.93-2.12) | (0.46-0.87) | (0.75-1.46) | (0.76-1.76) | (0.73-1.75) | (0.57-1.12) | (0.83-1.96) | (0.48-1.17) | (0.74-1.76) |
| 55 – 59yrs | 1.64* | 0.71* | 2.56*** | 1.22 | 2.05*** | 0.72* | 1.26 | 1.30 | 1.12 | 0.78 | 1.28 | 0.61* | 1.21 |
|  | (1.08-2.47) | (0.51-0.99) | (1.73-3.81) | (0.91-1.64) | (1.39-3.01) | (0.54-0.95) | (0.94-1.69) | (0.99-1.92) | (0.74-1.68) | (0.57-1.08) | (0.86-1.92) | (0.40-0.93) | (0.81-1.82) |
| 60 - 64yrs | 1.62* | 0.89 | 2.49*** | 1.40* | 2.21*** | 0.79 | 1.31 | 1.51* | 1.25 | 0.96 | 1.46 | 0.82 | 1.38 |
|  | (1.07-2.44) | (0.64-1.24) | (1.68-3.70) | (1.04-1.87) | (1.51-3.24) | (0.60-1.04) | (0.98-1.75) | (1.03-2.23) | (0.84-1.87) | (0.73-1.27) | (0.98-2.18) | (0.54-1.23) | (0.92-2.06) |
| P value | ***0.314*** | ***<0.001*** | ***<0.001*** | ***0.003*** | ***<0.001*** | ***<0.001*** | ***0.233*** | ***0.178*** | ***0.682*** | ***<0.001*** | ***0.427*** | ***0.057*** | ***0.754*** |

Model uses weighted data adjusted for country, sex, age, education and ethnicity. Covariate p values are adjusted for multiple comparisons using a Bonferroni correction. AOR = Adjusted Odds Ratio. Statistically significant differences denoted by *p<0.05, **p<0.01, ***p<0.001. # denotes policies not measured in Canadian respondents aged 18 – 30 years (reference category for age variable was 30-34 years).

**Supplemental Table 3** con’t

|  | | | | | | | | | | | | | |
| --- | --- | --- | --- | --- | --- | --- | --- | --- | --- | --- | --- | --- | --- |
|  | Subsidies to reduce the price of fresh fruit and vegetables | Calorie amounts on menus of chain restaurants | A maximum limit on salt levels in pre-packaged foods | A ban on marketing unhealthy food and beverages to children | Water or milk as the default drink in children’s meals | Taxes on sugary drinks if the money was spent on subsidising healthy food | Taxes on sugary drinks | Restrictions on maximum size of single serve soft drink | Zoning to restrict the number of fast food restaurants near schools | Taxes on foods with high sugar | A ban on toys, vouchers and competitions in children’s fast food meals | Restriction on sponsorship of sporting events and teams by food companies | A ban on marketing all food and beverages to children |
|  | AOR  (95%CI) | AOR  (95%CI) | AOR  (95%CI) | AOR  (95%CI) | AOR  (95%CI) | AOR  (95%CI) | AOR  (95%CI) | AOR  (95%CI) | AOR  (95%CI) | AOR  (95%CI) | AOR  (95%CI)) | AOR  (95%CI) | AOR  (95%CI) |
| Education (Reference = Low) | | |  |  |  |  |  |  |  |  |  |  |  |
| Medium | 1.02 | 1.12 | 1.06 | 1.43** | 1.43** | 1.57*** | 1.54** | 1.37* | 1.54** | 1.54** | 1.34* | 1.23 | 1.55** |
|  | (0.74-1.40) | (0.85-1.48) | (0.79-1.43) | (1.10-1.86) | (1.09-1.88) | (1.22-2.02) | (1.18-2.01) | (1.04-1.81) | (1.16-2.05) | (1.15-2.06) | (1.01-1.78) | (0.91-1.67) | (1.16-2.06) |
| High | 0.74 | 1.34* | 0.93 | 1.48** | 1.33* | 1.76*** | 1.70*** | 1.21 | 1.32 | 1.64** | 1.42* | 1.09 | 1.47* |
|  | (0.54-1.02) | (1.01-1.77) | (0.69-1.25) | (1.14-1.92) | (1.01-1.76) | (1.37-2.25) | (1.31-2.21) | (0.91-1.61) | (0.99-1.78) | (1.22-2.21) | (1.07-1.90) | (0.80-1.49) | (1.10-1.97) |
| P value | ***0.022*** | ***0.078*** | ***0.523*** | ***0.009*** | ***0.031*** | ***<0.001*** | ***<0.001*** | ***0.077*** | ***0.011*** | ***0.004*** | ***0.050*** | ***0.336*** | ***0.010*** |
| Ethnicity (Reference = Majority) | | | | |  |  |  |  |  |  |  |  |  |
| Minority | 0.87 | 0.73** | 0.75* | 0.73** | 0.84 | 0.75** | 0.84 | 1.14 | 1.03 | 0.96 | 0.84 | 1.03 | 0.88 |
|  | (0.66-1.14) | (0.59-0.91) | (0.58-0.97) | (0.60-0.89) | (0.65-1.08) | (0.61-0.92) | (0.68-1.03) | (0.89-1.47) | (0.80-1.34) | (0.74-1.24) | (0.64-1.09) | (0.79-1.36) | (0.68-1.15) |
| P value | ***0.317*** | ***0.005*** | ***0.030*** | ***0.002*** | ***0.167*** | ***0.005*** | ***0.090*** | ***0.295*** | ***0.804*** | ***0.733*** | ***0.193*** | ***0.812*** | ***0.350*** |

Model uses weighted data adjusted for country, sex, age, education and ethnicity. Covariate p values are adjusted for multiple comparisons using a Bonferroni correction. AOR = Adjusted Odds Ratio. Statistically significant differences denoted by *p<0.05, **p<0.01, ***p<0.001.
