# Supplementary material for: A multi-country survey of public support for food policies to promote healthy diets: Findings from the International Food Policy Study
Source: BMC Public Health. 2019 Sep 2;19:1205. doi: 10.1186/s12889-019-7483-9 (PMC6721115; doi:10.1186/s12889-019-7483-9)
Supplement: Supplementary file 4 — Table S4. Results from logistic regression model for support of food policies among Mexican respondents (n = 4057) (DOCX 44 kb) [file 12889_2019_7483_MOESM4_ESM.docx]

Additional file 4: Table S4 Results from logistic regression model for support of food policies among Mexican respondents (n=4,057)

|  | Subsidies to reduce the price of fresh fruit and vegetables | Calorie amounts on menus of chain restaurants | A maximum limit on salt levels in pre-packaged foods | A ban on marketing unhealthy food and beverages to children | Water or milk as the default drink in children’s meals | Taxes on sugary drinks if the money was spent on subsidising healthy food | | Taxes on sugary drinks | | Restrictions on maximum size of single serve soft drink | | Zoning to restrict the number of fast food restaurants near schools | | Taxes on foods with high sugar | | A ban on toys, vouchers and competitions in children’s fast food meals | | Restriction on sponsorship of sporting events and teams by food companies | | A ban on marketing all food and beverages to children | |  |
| --- | --- | --- | --- | --- | --- | --- | --- | --- | --- | --- | --- | --- | --- | --- | --- | --- | --- | --- | --- | --- | --- | --- |
|  | AOR  (95%CI) | AOR  (95%CI) | AOR  (95%CI) | AOR  (95%CI) | AOR  (95%CI) | AOR  (95%CI) | | AOR  (95%CI) | | AOR  (95%CI) | | AOR  (95%CI) | | AOR  (95%CI) | | AOR  (95%CI)) | | AOR  (95%CI) | | AOR  (95%CI) | |  |
| Sex (Reference = Male) | | | | | | | | | | | | | | | | | | | | | |  |
| Female | 1.39*** | 1.20* | 1.09 | 1.08 | 1.02 | 1.04 | | 0.96 | | 1.15 | | 0.88 | | 1.01 | | 0.83* | | 0.91 | | 0.95 | |  |
|  | (1.16-1.66) | (1.02-1.41) | (0.92-1.28) | (0.92-1.26) | (0.87-1.20) | (0.89-1.21) | | (0.83-1.11) | | (0.99-1.33) | | (0.76-1.02) | | (0.87-1.17) | | (0.71-0.97) | | (0.79-1.06) | | (0.82-1.10) | |  |
| P value | ***<0.001*** | ***0.027*** | ***0.311*** | ***0.351*** | ***0.809*** | ***0.625*** | | ***0.584*** | | ***0.072*** | | ***0.101*** | | ***0.910*** | | ***0.016*** | | ***0.218*** | | ***0.491*** | |  |
| Age (Reference = 18-24yrs) | | | | | | | | | | | | | | | | | | | | | |  |
| 25 – 29yrs | 1.11 | 1.06 | 1.19 | 1.11 | 1.28* | 1.30* | | 1.19 | | 1.37** | | 1.07 | | 1.15 | | 1.27* | | 1.18 | | 1.21 | |  |
|  | (0.87-1.42) | (0.85-1.32) | (0.95-1.49) | (0.89-1.38) | (1.02-1.59) | (1.05-1.61) | | (0.97-1.47) | | (1.11-1.70) | | (0.87-1.32) | | (0.94-1.42) | | (1.02-1.58) | | (0.95-1.45) | | (0.97-1.50) | |  |
| 30 – 34yrs | 1.10 | 1.31* | 1.11 | 1.30* | 1.31* | 1.30* | | 1.09 | | 1.11 | | 1.04 | | 1.11 | | 1.38* | | 1.20 | | 1.33* | |  |
|  | (0.84-1.45) | (1.02-1.70) | (0.86-1.44) | (1.02-1.67) | (1.01-1.68) | (1.02-1.66) | | (0.86-1.38) | | (0.88-1.41) | | (0.82-1.32) | | (0.88-1.40) | | (1.08-1.76) | | (0.95-1.52) | | (1.04-1.70) | |  |
| 35 – 39yrs | 1.05 | 1.43* | 1.23 | 1.28 | 1.17 | 1.30* | | 0.97 | | 1.09 | | 0.92 | | 1.05 | | 1.32* | | 1.23 | | 1.21 | |  |
|  | (0.79-1.39) | (1.09-1.88) | (0.94-1.61) | (0.99-1.65) | (0.90-1.52) | (1.02-1.68) | | (0.76-1.24) | | (0.85-1.39) | | (0.72-1.18) | | (0.82-1.33) | | (1.02-1.69) | | (0.96-1.58) | | (0.94-1.56) | |  |
| 40 – 44yrs | 1.80** | 1.57** | 1.48* | 1.76*** | 1.80*** | 1.45* | | 1.37* | | 0.95 | | 1.17 | | 1.32* | | 1.64*** | | 1.37* | | 1.45** | |  |
|  | (1.26-2.58) | (1.15-2.15) | (1.08-2.01) | (1.31-2.39) | (1.31-2.46) | (1.09-1.93) | | (1.04-1.81) | | (0.72-1.25) | | (0.89-1.54) | | (1.01-1.74) | | (1.24-2.16) | | (1.04-1.81) | | (1.10-1.92) | |  |
| 45 – 49yrs | 1.05 | 1.44* | 1.71** | 1.45* | 1.61** | 1.30 | | 1.25 | | 1.08 | | 0.99 | | 1.32 | | 1.59** | | 1.30 | | 1.30 | |  |
|  | (0.75-1.47) | (1.04-1.99) | (1.22-2.40) | (1.07-1.97) | (1.16-2.22) | (0.97-1.74) | | (0.94-1.67) | | (0.81-1.44) | | (0.74-1.32) | | (0.99-1.75) | | (1.19-2.13) | | (0.98-1.73) | | (0.97-1.74) | |  |
| 50 – 54yrs | 1.95** | 2.20*** | 1.96** | 2.19*** | 2.33*** | 2.01*** | | 1.46* | | 1.34 | | 1.49* | | 1.35 | | 1.90*** | | 1.50* | | 1.60** | |  |
|  | (1.24-3.06) | (1.45-3.36) | (1.29-2.98) | (1.47-3.27) | (1.55-3.51) | (1.38-2.92) | | (1.03-2.07) | | (0.94-1.90) | | (1.06-2.11) | | (0.96-1.91) | | (1.34-2.69) | | (1.06-2.13) | | (1.13-2.27) | |  |
| 55 – 59yrs | 2.04** | 3.84*** | 2.67*** | 2.30** | 3.72*** | 2.21** | | 1.64* | | 1.26 | | 1.03 | | 1.62* | | 2.79*** | | 1.34 | | 1.38 | |  |
|  | (1.21-3.44) | (2.13-6.92) | (1.57-4.55) | (1.42-3.72) | (2.07-6.69) | (1.40-3.48) | | (1.09-2.47) | | (0.83-1.90) | | (0.69-1.54) | | (1.08-2.44) | | (1.85-4.20) | | (0.89-2.02) | | (0.92-2.08) | |  |
| 60 - 64yrs | 1.21 | 3.45*** | 1.98* | 2.40** | 1.85* | 2.91*** | | 2.06** | | 1.25 | | 1.72* | | 1.62 | | 2.39*** | | 1.51 | | 1.87** | |  |
|  | (0.68-2.16) | (1.75-6.80) | (1.10-3.55) | (1.34-4.31) | (1.06-3.24) | (1.68-5.05) | | (1.24-3.41) | | (0.77-2.04) | | (1.07-2.78) | | (0.99-2.65) | | (1.49-3.86) | | (0.94-2.42) | | (1.17-2.98) | |  |
| P value | ***0.004*** | ***<0.001*** | ***<0.001*** | ***<0.001*** | ***<0.001*** | | ***<0.001*** | | ***0.007*** | | ***0.120*** | | ***0.123*** | | ***0.095*** | | ***<0.001*** | | ***0.203*** | | ***0.040*** | |

Model uses weighted data adjusted for country, sex, age, education and ethnicity. Covariate p values are adjusted for multiple comparisons using a Bonferroni correction. AOR = Adjusted Odds Ratio. Statistically significant differences denoted by *p<0.05, **p<0.01, ***p<0.001.

|  | | | | | | | | | | | | | |
| --- | --- | --- | --- | --- | --- | --- | --- | --- | --- | --- | --- | --- | --- |
|  | Subsidies to reduce the price of fresh fruit and vegetables | Calorie amounts on menus of chain restaurants | A maximum limit on salt levels in pre-packaged foods | A ban on marketing unhealthy food and beverages to children | Water or milk as the default drink in children’s meals | Taxes on sugary drinks if the money was spent on subsidising healthy food | Taxes on sugary drinks | Restrictions on maximum size of single serve soft drink | Zoning to restrict the number of fast food restaurants near schools | Taxes on foods with high sugar | A ban on toys, vouchers and competitions in children’s fast food meals | Restriction on sponsorship of sporting events and teams by food companies | A ban on marketing all food and beverages to children |
|  | AOR  (95%CI) | AOR  (95%CI) | AOR  (95%CI) | AOR  (95%CI) | AOR  (95%CI) | AOR  (95%CI) | AOR  (95%CI) | AOR  (95%CI) | AOR  (95%CI) | AOR  (95%CI) | AOR  (95%CI)) | AOR  (95%CI) | AOR  (95%CI) |
| Education (Reference = Low) | | |  |  |  |  |  |  |  |  |  |  |  |
| Medium | 0.90 | 0.85 | 0.85 | 0.94 | 0.80 | 0.72* | 0.90 | 0.82 | 0.82 | 0.87 | 0.90 | 0.84 | 0.98 |
|  | (0.66-1.23) | (0.64-1.12) | (0.64-1.13) | (0.71-1.25) | (0.60-1.06) | (0.55-0.95) | (0.69-1.18) | (0.62-1.07) | (0.62-1.07) | (0.67-1.14) | (0.68-1.20) | (0.64-1.10) | (0.74-1.30) |
| High | 1.02 | 1.21 | 1.14 | 1.21 | 1.03 | 1.00 | 1.19 | 1.11 | 1.03 | 1.16 | 1.27* | 1.01 | 1.27* |
|  | (0.80-1.29) | (0.98-1.51) | (0.92-1.42) | (0.98-1.49) | (0.83-1.28) | (0.82-1.23) | (0.97-1.45) | (0.91-1.36) | (0.84-1.26) | (0.95-1.41) | (1.03-1.56) | (0.83-1.24) | (1.03-1.56) |
| P value | ***0.667*** | ***0.010*** | ***0.056*** | ***0.062*** | ***0.138*** | ***0.023*** | ***0.038*** | ***0.032*** | ***0.157*** | ***0.037*** | ***0.007*** | ***0.295*** | ***0.026*** |
| Ethnicity (Reference = Majority) | | | | | | | | | | | | | |
| Minority | 0.82 | 0.75* | 0.81 | 0.97 | 0.95 | 0.93 | 1.15 | 1.06 | 1.25* | 1.01 | 1.29* | 1.28* | 1.16 |
|  | (0.64-1.05) | (0.59-0.94) | (0.64-1.02) | (0.77-1.23) | (0.75-1.20) | (0.74-1.16) | (0.92-1.43) | (0.85-1.32) | (1.01-1.56) | (0.81-1.25) | (1.04-1.61) | (1.03-1.59) | (0.93-1.45) |
| P value | ***0.119*** | ***0.013*** | ***0.079*** | ***0.826*** | ***0.645*** | ***0.517*** | ***0.217*** | ***0.619*** | ***0.043*** | ***0.964*** | ***0.021*** | ***0.028*** | ***0.190*** |

**Supplemental Table 4** con’t

Model uses weighted data adjusted for country, sex, age, education and ethnicity. Covariate p values are adjusted for multiple comparisons using a Bonferroni correction. AOR = Adjusted Odds Ratio. Statistically significant differences denoted by *p<0.05, **p<0.01, ***p<0.001.
