# Supplementary material for: A multi-country survey of public support for food policies to promote healthy diets: Findings from the International Food Policy Study
Source: BMC Public Health. 2019 Sep 2;19:1205. doi: 10.1186/s12889-019-7483-9 (PMC6721115; doi:10.1186/s12889-019-7483-9)
Supplement: Supplementary file 5 — Table S5. Results from logistic regression model for support of food policies among UK respondents (n = 4047) (DOCX 29 kb) [file 12889_2019_7483_MOESM5_ESM.docx]

**Additional file 5: Table S5** Results from logistic regression model for support of food policies among UK respondents (n=4,047)

|  | Subsidies to reduce the price of fresh fruit and vegetables | Calorie amounts on menus of chain restaurants | A maximum limit on salt levels in pre-packaged foods | A ban on marketing unhealthy food and beverages to children | Water or milk as the default drink in children’s meals | Taxes on sugary drinks if the money was spent on subsidising healthy food | Taxes on sugary drinks | Restrictions on maximum size of single serve soft drink | Zoning to restrict the number of fast food restaurants near schools | Taxes on foods with high sugar | A ban on toys, vouchers and competitions in children’s fast food meals | Restriction on sponsorship of sporting events and teams by food companies | A ban on marketing all food and beverages to children |
| --- | --- | --- | --- | --- | --- | --- | --- | --- | --- | --- | --- | --- | --- |
|  | AOR  (95%CI) | AOR  (95%CI) | AOR  (95%CI) | AOR  (95%CI) | AOR  (95%CI) | AOR  (95%CI) | AOR  (95%CI) | AOR  (95%CI) | AOR  (95%CI) | AOR  (95%CI) | AOR  (95%CI)) | AOR  (95%CI) | AOR  (95%CI) |
| Sex (Reference = Male) | | | | | | | | | | | | | |
| Female | 1.71*** | 1.30** | 1.43*** | 1.22* | 1.31** | 1.21* | 1.07 | 1.42*** | 1.25** | 1.02 | 1.06 | 1.24** | 1.20* |
|  | (1.45-2.01) | (1.11-1.52) | (1.22-1.68) | (1.04-1.43) | (1.12-1.53) | (1.04-1.41) | (0.92-1.25) | (1.21-1.66) | (1.07-1.46) | (0.88-1.19) | (0.90-1.24) | (1.06-1.45) | (1.03-1.41) |
| P value | ***<0.001*** | ***0.001*** | ***<0.001*** | ***0.015*** | ***0.001*** | ***0.012*** | ***0.381*** | ***<0.001*** | ***0.005*** | ***0.785*** | ***0.497*** | ***0.006*** | ***0.023*** |
| Age (Reference = 18-24yrs) | | | | | | | | | | | | | |
| 25 – 29yrs | 1.31* | 1.12 | 1.34* | 1.32* | 1.01 | 1.15 | 1.06 | 1.08 | 1.41** | 1.16 | 1.12 | 1.10 | 1.22 |
|  | (1.03-1.65) | (0.89-1.41) | (1.06-1.68) | (1.05-1.66) | (0.80-1.27) | (0.93-1.44) | (0.84-1.34) | (0.85-1.38) | (1.11-1.78) | (0.93-1.46) | (0.87-1.42) | (0.87-1.39) | (0.95-1.57) |
| 30 – 34yrs | 1.20 | 1.32 | 1.48** | 1.46* | 0.98 | 1.01 | 1.26 | 1.45* | 1.70*** | 1.23 | 1.44* | 1.16 | 1.67** |
|  | (0.89-1.61) | (0.99-1.76) | (1.11-1.98) | (1.09-1.94) | (0.74-1.30) | (0.76-1.33) | (0.94-1.68) | (1.08-1.95) | (1.27-2.28) | (0.93-1.64) | (1.07-1.95) | (0.86-1.55) | (1.23-2.27) |
| 35 – 39yrs | 1.44* | 1.49** | 1.90*** | 1.66** | 1.25 | 1.12 | 1.12 | 1.44* | 1.57** | 1.32 | 1.68** | 1.28 | 1.75** |
|  | (1.03-2.02) | (1.08-2.07) | (1.36-2.64) | (1.21-2.30) | (0.91-1.73) | (0.82-1.53) | (0.82-1.55) | (1.03-2.00) | (1.14-2.17) | (0.96-1.81) | (1.21-2.35) | (0.93-1.78) | (1.25-2.45) |
| 40 – 44yrs | 1.42* | 1.35 | 2.03*** | 1.67** | 1.10 | 1.15 | 1.56** | 1.41* | 1.86*** | 1.53** | 1.85*** | 1.22 | 1.54* |
|  | (1.01-1.99) | (0.97-1.87) | (1.45-2.85) | (1.20-2.32) | (0.79-1.53) | (0.84-1.57) | (1.12-2.16) | (1.01-1.98) | (1.34-2.59) | (1.11-2.11) | (1.33-2.59) | (0.88-1.70) | (1.09-2.18) |
| 45 – 49yrs | 1.77** | 1.40* | 2.13*** | 2.14*** | 1.25 | 1.73** | 1.37 | 1.31 | 2.20*** | 1.49* | 2.23*** | 1.67** | 1.91*** |
|  | (1.27-2.48) | (1.01-1.93) | (1.53-2.95) | (1.55-2.96) | (0.91-1.71) | (1.26-2.37) | (0.99-1.89) | (0.04-1.83) | (1.59-3.04) | (1.09-2.06) | (1.60-3.12) | (1.20-2.30) | (1.36-2.68) |
| 50 – 54yrs | 2.50*** | 1.96*** | 3.83*** | 2.38*** | 1.70** | 1.50** | 1.70** | 1.51* | 2.23*** | 1.96*** | 2.57*** | 1.38* | 1.90*** |
|  | (1.78-3.52) | (1.42-2.70) | (2.72-5.38) | (1.71-3.28) | (1.25-2.33) | (1.11-2.03) | (1.25-2.33) | (1.10-2.09) | (1.63-3.07) | (1.44-2.67) | (1.87-3.53) | (1.01-1.90) | (1.37-2.63) |
| 55 – 59yrs | 2.10*** | 2.14*** | 2.50*** | 2.96*** | 1.65** | 1.89*** | 2.32*** | 1.64** | 3.34*** | 2.05*** | 2.28*** | 1.58** | 1.83*** |
|  | (1.49-2.97) | (1.54-2.98) | (1.80-3.48) | (2.12-4.14) | (1.20-2.27) | (1.39-2.58) | (1.68-3.19) | (1.19-2.26) | (1.70-3.22) | (1.51-2.80) | (1.65-3.14) | (1.15-2.18) | (1.32-2.54) |
| 60 - 64yrs | 2.31*** | 2.31*** | 5.09*** | 4.03*** | 2.27*** | 3.07*** | 3.25*** | 2.41*** | 3.28*** | 3.20*** | 3.57*** | 1.72** | 1.89*** |
|  | (1.64-3.27) | (1.67-3.21) | (3.60-7.20) | (2.85-5.71) | (1.65-3.10) | (2.24-4.21) | (2.36-4.47) | (1.76-3.31) | (2.37-4.53) | (2.35-4.35) | (2.59-4.91) | (1.26-2.37) | (1.36-2.61) |
| P value | ***<0.001*** | ***<0.001*** | ***<0.001*** | ***<0.001*** | ***<0.001*** | ***<0.001*** | ***<0.001*** | ***<0.001*** | ***<0.001*** | ***<0.001*** | ***<0.001*** | ***0.003*** | ***<0.001*** |

Model uses weighted data adjusted for country, sex, age, education and ethnicity. Covariate p values are adjusted for multiple comparisons using a Bonferroni correction. AOR = Adjusted Odds Ratio. Statistically significant differences denoted by *p<0.05, **p<0.01, ***p<0.001.

**Supplemental Table 5** con’t

|  | Subsidies to reduce the price of fresh fruit and vegetables | Calorie amounts on menus of chain restaurants | A maximum limit on salt levels in pre-packaged foods | A ban on marketing unhealthy food and beverages to children | Water or milk as the default drink in children’s meals | Taxes on sugary drinks if the money was spent on subsidising healthy food | Taxes on sugary drinks | Restrictions on maximum size of single serve soft drink | Zoning to restrict the number of fast food restaurants near schools | Taxes on foods with high sugar | A ban on toys, vouchers and competitions in children’s fast food meals | Restriction on sponsorship of sporting events and teams by food companies | A ban on marketing all food and beverages to children |
| --- | --- | --- | --- | --- | --- | --- | --- | --- | --- | --- | --- | --- | --- |
|  | AOR  (95%CI) | AOR  (95%CI) | AOR  (95%CI) | AOR  (95%CI) | AOR  (95%CI) | AOR  (95%CI) | AOR  (95%CI) | AOR  (95%CI) | AOR  (95%CI) | AOR  (95%CI) | AOR  (95%CI)) | AOR  (95%CI) | AOR  (95%CI) |
| Education (Reference = Low) | | |  |  |  |  |  |  |  |  |  |  |  |
| Medium | 1.40** | 1.43** | 1.35** | 1.61*** | 1.32* | 1.49*** | 1.67*** | 1.25 | 1.25* | 1.36** | 1.37** | 1.25* | 1.27* |
|  | (1.12-1.76) | (1.15-1.78) | (1.09-1.69) | (1.29-2.01) | (1.07-1.64) | (1.21-1.83) | (1.34-2.07) | (1.00-1.56) | (1.01-1.56) | (1.10-1.68) | (1.10-1.71) | (1.00-1.56) | (1.01-1.59) |
| High | 1.27* | 1.50*** | 1.41** | 1.78*** | 1.32** | 1.84*** | 1.86*** | 1.36** | 1.38** | 1.54*** | 1.37** | 1.44*** | 1.14 |
|  | (1.04-1.56) | (1.23-1.83) | (1.15-1.73) | (1.46-2.17) | (1.09-1.60) | (1.52-2.22) | (1.53-2.27) | (1.11-1.66) | (1.14-1.69) | (1.27-1.86) | (1.12-1.68) | (1.18-1.76) | (0.93-1.41) |
| P value | ***0.010*** | ***<0.001*** | ***0.003*** | ***<0.001*** | ***0.011*** | ***<0.001*** | ***<0.001*** | ***0.011*** | ***0.005*** | ***<0.001*** | ***0.005*** | ***0.001*** | ***0.127*** |
| Ethnicity (Reference = Majority) | | | | | | | | | | | | | |
| Minority | 0.84 | 0.71** | 0.78 | 1.04 | 0.85 | 0.85 | 0.85 | 1.11 | 1.15 | 1.01 | 1.09 | 1.13 | 1.04 |
|  | (0.65-1.08) | (0.55-0.92) | (0.60-1.01) | (0.80-1.35) | (0.66-1.09) | (0.67-1.09) | (0.66-1.09) | (0.86-1.43) | (0.89-1.50) | (0.79-1.29) | (0.83-1.42) | (0.87-1.46) | (0.79-1.38) |
| P value | ***0.179*** | ***0.009*** | ***0.056*** | ***0.751*** | ***0.202*** | ***0.202*** | ***0.203*** | ***0.438*** | ***0.281*** | ***0.924*** | ***0.550*** | ***0.365*** | ***0.764*** |

Model uses weighted data adjusted for country, sex, age, education and ethnicity. Covariate p values are adjusted for multiple comparisons using a Bonferroni correction. AOR = Adjusted Odds Ratio. Statistically significant differences denoted by *p<0.05, **p<0.01, ***p<0.001.
