# Supplementary material for: A multi-country survey of public support for food policies to promote healthy diets: Findings from the International Food Policy Study
Source: BMC Public Health. 2019 Sep 2;19:1205. doi: 10.1186/s12889-019-7483-9 (PMC6721115; doi:10.1186/s12889-019-7483-9)
Supplement: Supplementary file 6 — Table S6. Results from logistic regression model for support of food policies among US respondents (n = 4868) (DOCX 45 kb) [file 12889_2019_7483_MOESM6_ESM.docx]

Additional file 6: Table S6 Results from logistic regression model for support of food policies among US respondents (n=4,868)

|  | Subsidies to reduce the price of fresh fruit and vegetables | Calorie amounts on menus of chain restaurants | A maximum limit on salt levels in pre-packaged foods | A ban on marketing unhealthy food and beverages to children | Water or milk as the default drink in children’s meals | Taxes on sugary drinks if the money was spent on subsidising healthy food | Taxes on sugary drinks | Restrictions on maximum size of single serve soft drink | Zoning to restrict the number of fast food restaurants near schools | Taxes on foods with high sugar | A ban on toys, vouchers and competitions in children’s fast food meals | Restriction on sponsorship of sporting events and teams by food companies | A ban on marketing all food and beverages to children |
| --- | --- | --- | --- | --- | --- | --- | --- | --- | --- | --- | --- | --- | --- |
|  | AOR  (95%CI) | AOR  (95%CI) | AOR  (95%CI) | AOR  (95%CI) | AOR  (95%CI) | AOR  (95%CI) | AOR  (95%CI) | AOR  (95%CI) | AOR  (95%CI) | AOR  (95%CI) | AOR  (95%CI)) | AOR  (95%CI) | AOR  (95%CI) |
| Sex (Reference = Male) | | | | | | | | | | | | | |
| Female | 2.13*** | 1.81*** | 1.89*** | 1.55*** | 1.75*** | 1.41*** | 1.26** | 1.79*** | 1.37*** | 1.28** | 1.41*** | 1.21* | 1.37*** |
|  | (1.84-2.47) | (1.56-2.10) | (1.64-2.18) | (1.35-1.60) | (1.52-2.03) | (1.22-1.64) | (1.08-1.47) | (1.53-2.10) | (1.16-1.61) | (1.10-1.51) | (1.20-1.67) | (1.02-1.44) | (1.15-1.62) |
| P value | ***<0.001*** | ***<0.001*** | ***<0.001*** | ***<0.001*** | ***<0.001*** | ***<0.001*** | ***0.003*** | ***<0.001*** | ***<0.001*** | ***0.002*** | ***<0.001*** | ***0.033*** | ***<0.001*** |
| Age (Reference = 18-24yrs) | | | | | | | | | | | | | |
| 25 – 29yrs | 1.38* | 0.93 | 1.25 | 1.25 | 0.98 | 1.33* | 1.26 | 1.06 | 1.03 | 1.16 | 1.10 | 1.13 | 1.32 |
|  | (1.07-1.79) | (0.72-1.20) | (0.98-1.61) | (0.98-1.60) | (0.77-1.25) | (1.04-1.70) | (0.96-1.64) | (0.82-1.37) | (0.80-1.34) | (0.89-1.51) | (0.83-1.45) | (0.85-1.50) | (0.98-1.77) |
| 30 – 34yrs | 1.07 | 0.81 | 1.03 | 1.31 | 0.84 | 1.10 | 1.24 | 1.07 | 1.08 | 1.04 | 1.22 | 1.29 | 1.48* |
|  | (0.79-1.46) | (0.60-1.10) | (0.76-1.38) | (0.98-1.77) | (0.62-1.13) | (0.82-1.47) | (0.90-1.70) | (0.79-1.46) | (0.79-1.48) | (0.76-1.42) | (0.88-1.70) | (0.92-1.80) | (1.06-2.08) |
| 35 – 39yrs | 1.17 | 0.85 | 1.12 | 1.48* | 1.10 | 1.19 | 1.33 | 1.26 | 1.18 | 1.43* | 1.27 | 1.43 | 1.39 |
|  | (0.84-1.64) | (0.61-1.18) | (0.81-1.55) | (1.07-2.04) | (0.80-1.52) | (0.86-1.64) | (0.94-1.86) | (0.90-1.76) | (0.84-1.66) | (1.02-2.00) | (0.89-1.80) | (1.00-2.05) | (0.96-2.02) |
| 40 – 44yrs | 0.97 | 0.90 | 1.03 | 1.30 | 1.02 | 0.96 | 0.99 | 0.94 | 1.07 | 0.89 | 0.99 | 1.25 | 1.41 |
|  | (0.68-1.40) | (0.62-1.30) | (0.72-1.48) | (0.90-1.87) | (0.72-1.45) | (0.67-1.37) | (0.68-1.46) | (0.65-1.37) | (0.73-1.57) | (0.60-1.31) | (0.66-1.48) | (0.84-1.88) | (0.93-2.14) |
| 45 – 49yrs | 1.00 | 1.02 | 1.31 | 1.04 | 0.99 | 0.91 | 0.94 | 0.91 | 0.90 | 0.95 | 1.27 | 1.12 | 1.28 |
|  | (0.71-1.42) | (0.72-1.45) | (0.93-1.83) | (0.74-1.46) | (0.71-1.38) | (0.65-1.28) | (0.65-1.35) | (0.64-1.29) | (0.63-1.29) | (0.66-1.36) | (0.88-1.83) | (0.76-1.64) | (0.86-1.89) |
| 50 – 54yrs | 0.82 | 0.87 | 1.36 | 1.27 | 0.83 | 1.05 | 1.02 | 0.59** | 0.64* | 0.82 | 0.79 | 0.93 | 1.14 |
|  | (0.58-1.15) | (0.62-1.22) | (0.97-1.91) | (0.91-1.78) | (0.59-1.15) | (0.75-1.48) | (0.71-1.47) | (0.41-0.85) | (0.44-0.93) | (0.57-1.20) | (0.53-1.18) | (0.63-1.39) | (0.76-1.70) |
| 55 – 59yrs | 0.86 | 1.11 | 1.19 | 1.30 | 1.01 | 0.80 | 0.88 | 0.55*** | 0.71* | 0.76 | 0.89 | 0.70* | 0.90 |
|  | (0.64-1.15) | (0.82-1.51) | (0.89-1.60) | (0.97-1.74) | (0.76-1.35) | (0.59-1.08) | (0.64-1.21) | (0.40-0.76) | (0.52-0.99) | (0.55-1.06) | (0.64-1.25) | (0.49-1.00) | (0.63-1.29) |
| 60 - 64yrs | 0.76 | 1.08 | 1.28 | 1.34* | 0.92 | 0.88 | 0.81 | 0.68* | 0.65** | 0.75 | 0.93 | 0.59** | 1.17 |
|  | (0.57-1.02) | (0.81-1.44) | (0.96-1.69) | (1.01-1.77) | (0.70-1.22) | (0.66-1.17) | (0.60-1.10) | (0.50-0.91) | (0.47-0.89) | (0.55-1.03) | (0.67-1.29) | (0.41-0.83) | (0.84-1.64) |
| P value | ***<0.001*** | ***0.384*** | ***0.371*** | ***0.358*** | ***0.702*** | ***<0.001*** | ***0.003*** | ***<0.001*** | ***<0.001*** | ***<0.001*** | ***0.127*** | ***<0.001*** | ***0.074*** |

Model uses weighted data adjusted for country, sex, age, education and ethnicity. Covariate p values are adjusted for multiple comparisons using a Bonferroni correction. AOR = Adjusted Odds Ratio. Statistically significant differences denoted by *p<0.05, **p<0.01, ***p<0.001.

**Supplemental Table 6** con’t

|  | | | | | | | | | | | | | |
| --- | --- | --- | --- | --- | --- | --- | --- | --- | --- | --- | --- | --- | --- |
|  | Subsidies to reduce the price of fresh fruit and vegetables | Calorie amounts on menus of chain restaurants | A maximum limit on salt levels in pre-packaged foods | A ban on marketing unhealthy food and beverages to children | Water or milk as the default drink in children’s meals | Taxes on sugary drinks if the money was spent on subsidising healthy food | Taxes on sugary drinks | Restrictions on maximum size of single serve soft drink | Zoning to restrict the number of fast food restaurants near schools | Taxes on foods with high sugar | A ban on toys, vouchers and competitions in children’s fast food meals | Restriction on sponsorship of sporting events and teams by food companies | A ban on marketing all food and beverages to children |
|  | AOR  (95%CI) | AOR  (95%CI) | AOR  (95%CI) | AOR  (95%CI) | AOR  (95%CI) | AOR  (95%CI) | AOR  (95%CI) | AOR  (95%CI) | AOR  (95%CI) | AOR  (95%CI) | AOR  (95%CI)) | AOR  (95%CI) | AOR  (95%CI) |
| Education (Reference = Low) | | |  |  |  |  |  |  |  |  |  |  |  |
| Medium | 0.89 | 1.23 | 1.23 | 1.15 | 0.94 | 1.25 | 1.44** | 1.06 | 1.14 | 1.43** | 1.29 | 1.26 | 1.40* |
|  | (0.70-1.13) | (0.97-1.55) | (0.98-1.55) | (0.91-1.44) | (0.74-1.18) | (0.99-1.59) | (1.12-1.87) | (0.82-1.36) | (0.88-1.47) | (1.10-1.86) | (1.00-1.68) | (0.95-1.66) | (1.06-1.85) |
| High | 0.89 | 1.28** | 1.07 | 1.13 | 1.02 | 1.59*** | 1.71*** | 1.12 | 1.18 | 1.67*** | 1.27* | 1.28* | 1.49** |
|  | (0.73-1.08) | (1.14-1.67) | (0.88-1.29) | (0.94-1.37) | (0.84-1.22) | (1.31-1.94) | (1.38-2.12) | (0.92-1.38) | (0.95-1.46) | (1.34-2.08) | (1.02-1.58) | (1.01-1.62) | (1.18-1.89) |
| P value | ***0.487*** | ***0.004*** | ***0.186*** | ***0.397*** | ***0.697*** | ***<0.001*** | ***<0.001*** | ***0.544*** | ***0.330*** | ***<0.001*** | ***0.077*** | ***0.120*** | ***0.004*** |
| Ethnicity (Reference = Majority) | | | | | | | | | | | | | |
| Minority | 1.09 | 0.91 | 1.40*** | 1.41*** | 1.38*** | 1.41*** | 1.41*** | 1.65*** | 1.86*** | 1.73*** | 1.60*** | 1.87*** | 1.45*** |
|  | (0.92-1.28) | (0.77-1.07) | (1.19-1.64) | (1.20-1.65) | (1.18-1.62) | (1.20-1.65) | (1.19-1.66) | (1.40-1.95) | (1.57-2.20) | (1.46-2.05) | (1.35-1.91) | (1.57-2.24) | (1.21-1.73) |
| P value | ***0.315*** | ***0.269*** | ***<0.001*** | ***<0.001*** | ***<0.001*** | ***<0.001*** | ***<0.001*** | ***<0.001*** | ***<0.001*** | ***<0.001*** | ***<0.001*** | ***<0.001*** | ***<0.001*** |

Model uses weighted data adjusted for country, sex, age, education and ethnicity. Covariate p values are adjusted for multiple comparisons using a Bonferroni correction. AOR = Adjusted Odds Ratio. Statistically significant differences denoted by *p<0.05, **p<0.01, ***p<0.001.
