# Supplementary material for: A multi-country survey of public support for food policies to promote healthy diets: Findings from the International Food Policy Study
Source: BMC Public Health. 2019 Sep 2;19:1205. doi: 10.1186/s12889-019-7483-9 (PMC6721115; doi:10.1186/s12889-019-7483-9)
Supplement: Supplementary file 7 — Table S7. Weighted proportion of ‘support’, ‘neutral’ and ‘oppose’ responses to each policy in the total sample and by country (DOCX 20 kb) [file 12889_2019_7483_MOESM7_ESM.docx]

**Additional file 7: Table S7** Weighted proportion of ‘support’, ‘neutral’ and ‘oppose’ responses to each policy in the total sample and by country.

|  | Total,  n=19,857 | | | Australia  n=3,767 | | | Canada  n=3,118 | | | Mexico  n=4,057 | | | UK  n=4,047 | | | US  n=4,868 | | |
| --- | --- | --- | --- | --- | --- | --- | --- | --- | --- | --- | --- | --- | --- | --- | --- | --- | --- | --- |
|  | Support  % | Neutral  % | Oppose  % | Support  % | Neutral  % | Oppose  % | Support  % | Neutral  % | Oppose  % | Support  % | Neutral  % | Oppose  % | Support  % | Neutral  % | Oppose  % | Support  % | Neutral  % | Oppose  % |
| Subsidies to reduce the price of fresh fruit and vegetables | 68.2 | 23.9 | 7.9 | 68.9 | 24 | 7.1 | 70.4 | 22.5 | 7.1 | 78.4 | 16.9 | 4.7 | 66.5 | 28.2 | 5.3 | 59.3 | 26.9 | 13.8 |
| Calorie amounts on menus of chain restaurants | 65.4 | 28.3 | 6.3 | 60.5 | 32.8 | 6.7 | 70.9 | 24.5 | 4.6 | 73.8 | 23.3 | 2.9 | 60.6 | 33.2 | 6.2 | 62.6 | 27.5 | 9.9 |
| Maximum limit on salt levels in pre-packaged foods | 61.4 | 28.7 | 9.9 | 60.4 | 29.7 | 9.9 | 64 | 28.2 | 7.8 | 73.6 | 22.3 | 4.1 | 64 | 29 | 7.0 | 48.5 | 33.2 | 18.3 |
| A ban on marketing unhealthy food and beverages to children | 57.2 | 31.2 | 11.6 | 56.9 | 32.1 | 11 | 61.4 | 29.2 | 9.4 | 68.1 | 25.9 | 6.0 | 59.4 | 32.4 | 8.2 | 43.5 | 35.4 | 21.1 |
| Requiring water or milk as the default drink in children’s fast food meal deals | 56.0 | 32.2 | 11.8 | 54.7 | 33.7 | 11.6 | 55.2 | 33.7 | 11.1 | 72.2 | 22.8 | 5.0 | 52.6 | 36.5 | 10.9 | 46.4 | 34.9 | 18.7 |
| Taxes on sugary drinks IF the money was spent on subsidising healthy food | 51.6 | 29.4 | 19.0 | 48.6 | 32.3 | 19.1 | 51.8 | 27.8 | 20.4 | 66.3 | 23.6 | 10.1 | 56.5 | 32.2 | 11.3 | 37.2 | 30.9 | 31.9 |
| Taxes on sugary drinks | 42.7 | 32.6 | 24.7 | 41.8 | 33.1 | 25.1 | 40.7 | 32 | 27.3 | 53.8 | 31.7 | 14.5 | 49.1 | 34.7 | 16.2 | 30 | 31.7 | 38.3 |
| Restrictions on maximum size (e.g., max of 375mL) of single serve soft drink | 42.3 | 36.5 | 21.2 | 43.3 | 37.2 | 19.5 | 40.9 | 37.9 | 21.2 | 56.6 | 33.5 | 9.9 | 41 | 40.3 | 18.7 | 31 | 34.7 | 34.3 |
| Zoning to restrict the number of fast food restaurants near schools | 41.2 | 39.9 | 18.9 | 44.4 | 39.2 | 16.4 | 35.7 | 43.1 | 21.2 | 49.3 | 40.5 | 10.2 | 48.4 | 39 | 12.6 | 28.3 | 39.2 | 32.5 |
| Taxes on foods with high sugar | 41.0 | 33.4 | 25.6 | 38.5 | 35.6 | 25.9 | 36.1 | 35.1 | 28.8 | 55.8 | 29.5 | 14.7 | 46.5 | 36.5 | 17 | 27.9 | 31.8 | 40.3 |
| A ban on toys, vouchers and competitions as part of children’s meals at fast food restaurants | 37.9 | 42.5 | 19.6 | 42.9 | 39.3 | 17.8 | 36.2 | 44.3 | 19.5 | 43.8 | 44.1 | 12.1 | 42.8 | 42.1 | 15.1 | 25.9 | 42.9 | 31.2 |
| Restriction on sponsorship of sporting events and teams by food companies such as Coca Cola and McDonalds | 37.3 | 40.9 | 21.8 | 39.8 | 39.8 | 20.4 | 29.7 | 42.8 | 27.5 | 49.5 | 39.6 | 10.9 | 43.4 | 42.0 | 14.6 | 23.3 | 41.3 | 35.4 |
| A ban on marketing all food and beverages to children | 34.7 | 42.7 | 22.6 | 37.8 | 41.5 | 20.7 | 36.1 | 42.0 | 21.9 | 40.6 | 44.4 | 15.0 | 37.8 | 43.7 | 18.5 | 24.0 | 42.0 | 34.0 |

Sample weights constructed using population estimates from the census in each country based on age group, gender and region.
